# Astrocyte reactivity is associated with tau tangle load and cortical thinning in Alzheimer’s disease

**DOI:** 10.1186/s13024-024-00750-8

**Published:** 2024-07-30

**Authors:** Tengfei Guo, Anqi Li, Pan Sun, Zhengbo He, Yue Cai, Guoyu Lan, Lin Liu, Jieyin Li, Jie Yang, Yalin Zhu, Ruiyue Zhao, Xuhui Chen, Dai Shi, Zhen Liu, Qingyong Wang, Linsen Xu, Liemin Zhou, Pengcheng Ran, Xinlu Wang, Kun Sun, Jie Lu, Ying Han

**Affiliations:** 1 Institute of Biomedical Engineering, Shenzhen Bay Laboratory, No.5 Kelian Road, Shenzhen, 518132 China; 2 Institute of Biomedical Engineering, Peking University Shenzhen Graduate School, Shenzhen, 518055 China; 3 Department of Neurology, Xuanwu Hospital of Capital Medical University, #45 Changchun Street, Xicheng District, Beijing, 100053 China; 4 Department of Nuclear Medicine, The First Affiliated Hospital, Guangzhou Medical University, Guangzhou, 510120 China; 5 Department of Neurology, Peking University Shenzhen Hospital, Shenzhen, 518000 China; 6 Neurology Medicine Center, The Seventh Affiliated Hospital, Sun Yat-sen University, Shenzhen, 518000 China; 7 Department of Neurology, Shenzhen Guangming District People’s Hospital, Shenzhen, 518107 China; 8 Department of Medical Imaging, Shenzhen Guangming District People’s Hospital, Shenzhen, 518106 China; 9 Department of Nuclear Medicine, Guangdong Hospital of Traditional Chinese Medicine, Guangzhou, 510120 China; 10 Institute of Cancer Research, Shenzhen Bay Laboratory, Shenzhen, 518132 China; 11 School of Biomedical Engineering, Hainan University, Haikou, 570228 China; 12 Center of Alzheimer’s Disease, Beijing Institute for Brain Disorders, Beijing, 100053 China; 13 National Clinical Research Center for Geriatric Diseases, Beijing, 100053 China; 14 Department of Radiology and Nuclear Medicine, Xuanwu Hospital, Capital Medical University, #45 Changchun Street, Xicheng District, Beijing, 100053 China

**Keywords:** Plasma biomarkers, Aβ, Tau, GFAP, Astrocyte reactivity, Alzheimer’s disease

## Abstract

**Background:**

It is not fully established whether plasma β-amyloid(Aβ)_42_/Aβ_40_ and phosphorylated Tau_181_ (p-Tau_181_) can effectively detect Alzheimer’s disease (AD) pathophysiology in older Chinese adults and how these biomarkers correlate with astrocyte reactivity, Aβ plaque deposition, tau tangle aggregation, and neurodegeneration.

**Methods:**

We recruited 470 older adults and analyzed plasma Aβ_42_/Aβ_40_, p-Tau_181_, glial fibrillary acidic protein (GFAP), and neurofilament light (NfL) using the Simoa platform. Among them, 301, 195, and 70 underwent magnetic resonance imaging, Aβ and tau positron emission tomography imaging. The plasma Aβ_42_/Aβ_40_ and p-Tau_181_ thresholds were defined as ≤0.0609 and ≥2.418 based on the receiver operating characteristic curve analysis using the Youden index by comparing Aβ-PET negative cognitively unimpaired individuals and Aβ-PET positive cognitively impaired patients. To evaluate the feasibility of using plasma Aβ_42_/Aβ_40_ (A) and p-Tau_181_ (T) to detect AD and understand how astrocyte reactivity affects this process, we compared plasma GFAP, Aβ plaque, tau tangle, plasma NfL, hippocampal volume, and temporal-metaROI cortical thickness between different plasma A/T profiles and explored their relations with each other using general linear models, including age, sex, *APOE-ε4*, and diagnosis as covariates.

**Results:**

Plasma A+/T + individuals showed the highest levels of astrocyte reactivity, Aβ plaque, tau tangle, and axonal degeneration, and the lowest hippocampal volume and temporal-metaROI cortical thickness. Lower plasma Aβ_42_/Aβ_40_ and higher plasma p-Tau_181_ were independently and synergistically correlated with higher plasma GFAP and Aβ plaque. Elevated plasma p-Tau_181_ and GFAP concentrations were directly and interactively associated with more tau tangle formation. Regarding neurodegeneration, higher plasma p-Tau_181_ and GFAP concentrations strongly correlated with more axonal degeneration, as measured by plasma NfL, and lower plasma Aβ_42_/Aβ_40_ and higher plasma p-Tau_181_ were related to greater hippocampal atrophy. Higher plasma GFAP levels were associated with thinner cortical thickness and significantly interacted with lower plasma Aβ_42_/Aβ_40_ and higher plasma p-Tau_181_ in predicting more temporal-metaROI cortical thinning. Voxel-wise imaging analysis confirmed these findings.

**Discussion:**

This study provides a valuable reference for using plasma biomarkers to detect AD in the Chinese community population and offers novel insights into how astrocyte reactivity contributes to AD progression, highlighting the importance of targeting reactive astrogliosis to prevent AD.

**Supplementary Information:**

The online version contains supplementary material available at 10.1186/s13024-024-00750-8.

## Background

Alzheimer’s disease (AD) patients exhibit reduced concentrations of β-amyloid (Aβ)42(Aβ_42_) [1–3] in CSF or plasma and elevated cortical Aβ accumulation [4, 5]. This is followed by increased levels of phosphorylated Tau (p-Tau) in CSF or plasma [6] and cortical tau tangles [7, 8], ultimately leading to neurodegeneration and cognitive decline [9–11]. Recently, neuroinflammation has been strongly linked to AD progression, providing additional pathological insights to predict AD core pathologies and neurodegeneration [12]. Understanding the association between neuroinflammation, Aβ, tau, and neurodegeneration is critical for comprehending AD’s characteristics and progression patterns.

Positron emission tomography (PET) imaging [9, 13] and CSF biomarkers [1, 6] are commonly used to detect abnormal alterations in AD pathologies. Previous studies using CSF biomarker [14, 15] and PET imaging [16–18] have demonstrated that individuals who are Aβ positive and tau positive (A+/T+) are at higher risk of AD than those who are Aβ negative and tau negative (A-/T-), Aβ positive and tau negative (A+/T-), and Aβ negative and tau positive (A-/T+). However, the high cost and limited availability of PET imaging and the invasiveness of lumbar puncture restrict their use in AD diagnosis. Advanced techniques for detecting plasma Aβ_42_/Aβ_40_ [19–21], p-Tau [22–28], astrocyte reactivity [29–31], and axonal degeneration [32, 33] suggest plasma biomarkers have great potential for diagnosing AD [34, 35].

Recently, plasma biomarker studies [36–40] and the latest NIA-AA research framework proposed by Jack and colleagues in AAIC suggest using a combination of plasma Aβ_42_/Aβ_40_ and plasma p-Tau, rather than each alone, to identify individuals at a high risk of AD. However, reliable thresholds for plasma A_β42_/Aβ_40_ and p-Tau_181_ have not been established in older adults in the Chinese community. It remains unclear whether A+/T + individuals defined by plasma biomarkers have widespread cortical Aβ plaque, tau tangle, hippocampal atrophy, and cortical thinning compared to the plasma A-/T- individuals in a community-based aging cohort, particularly in China. Furthermore, plasma glial fibrillary acidic protein (GFAP) has emerged as a promising biomarker for representing astrocyte reactivity [30, 31, 41–43]. Recent studies [29, 44] have shown that plasma GFAP may affect the association between Aβ and tau. However, the associations among plasma Aβ_42_/Aβ_40_, plasma p-Tau_181_, and plasma GFAP, as well as their independent and synergistic relationships to Aβ plaque accumulation, tau tangle aggregation, axonal degeneration, hippocampal atrophy, and AD-signature cortical thinning, remain unclear.

In this study, we analyzed plasma biomarkers, structural MRI, Aβ PET, and tau PET images based on a Chinese community-based aging cohort to (1) determine the effective thresholds for plasma Aβ_42_/Aβ_40_ and plasma p-Tau_181_ in older Chinese adults and evaluate their feasibility for detecting the abnormal alternations in astrocyte reactivity, Aβ plaque, tau tangle, axonal degeneration, hippocampal atrophy, and cortical thinning; (2) and investigate the associations among plasma Aβ_42_/Aβ_40_, plasma p-Tau_181_, and plasma GFAP, as well as how they independently and synergistically relate to Aβ plaque, tau tangle, axonal degeneration, hippocampal atrophy, and AD-signature cortical thinning in older adults. The ultimate goal of this study is to provide evidence of the feasibility of using plasma Aβ_42_/Aβ_40_ and plasma p-Tau_181_ to identify individuals at high risk of AD in the community and to determine whether elevated plasma GFAP concentrations can further predict AD downstream events.

## Methods

### Participants

The community-based longitudinal cohort Greater-Bay-Area Healthy Aging Brain Study (GHABS) [45](clinicaltrials.gov ID: NCT06183658) was approved by the Shenzhen Bay Laboratory and collaborated hospitals’ Ethical Committees, and was launched in May 2021. The written informed consent of the GHABS project was signed by each participant before enrollment. All the volunteer participants from the community in Guangdong-Hong Kong-Macao Greater-Bay-Area of South China underwent cognitive assessments, genetic screening, and blood sample collection, and part of them had MRI, Aβ PET, and tau PET scanning. The inclusion and exclusion criteria of the GHABS cohort were provided in the Supplementary Material. Participants were classified as cognitively unimpaired (CU), mild cognitive impairment (MCI), and AD dementia following the standard protocol of the ADNI cohort [46]. We analyzed 470 GHABS participants who simultaneously completed cognitive assessments, plasma Aβ_42_/Aβ_40_, p-Tau_181_, NfL, and GFAP data measured by the Simoa platform. Among them, 326, 87, and 57 were CU, MCI, and dementia. MCI and dementia patients were pooled as cognitively impaired (CI) individuals.

### Plasma biomarkers and APOE genotyping

Participants fasted for one night the day before (not less than 6 h), and blood was drawn in the morning of the next day. The details of plasma sample processing can be found in *Supplementary Material*. The concentrations of Aβ_40_, Aβ_42_, NfL, GFAP, and p-Tau_181_ in plasma were detected using commercial Simoa^®^ NEUROLOGY 4-PLEX E (N4PE, cat: 103670), and pTau-181 (cat: 104111) in Simoa HD-X Analyzer™ (Quanterix Corp.) in Shenzhen Bay Laboratory. APOE genotype was determined by TaqMan™ SNP genotyping for the two single nucleotide polymorphisms (rs429358, rs7412) that detect the ε2, ε3, and ε4 alleles using the DNA Isolation Kit based on the blood cell by centrifuge from the EDTA blood sample.

### MRI and PET imaging

All the MRI scanning sequences were conducted following the standard ADNI protocol. The 3 Plane Localizer positioning sequence and 3D T1 MPRAGE/IRSPGR MRI image data were collected on 3.0T scanners. The structural MRI images were segmented into different cortical and subcortical regions of interest (ROI) in Freesurfer (V7.2.0). The residual hippocampal volume (rHCV) was calculated using the hippocampal volume of both hemispheres and adjusted using the estimated total intracranial volume as we described previously [9]. In addition, the cortical thickness of AD-signature atrophy brain regions was obtained by calculating the surface area-weighted average thickness of the bilateral entorhinal, fusiform, inferior temporal, and middle temporal cortices [47].

The Aβ PET radiotracer [^18^F]D3FSP (FSP) [48] and tau PET radiotracer [^18^F]-flortaucipir (FTP) [49] were used for Aβ PET and tau PET imaging respectively. The Aβ PET and tau PET data acquisition were performed on a GE Discovery™ MI Gen 2 PET/CT scanner and a Siemens Biograph™ TruePoint™ PET/CT scanner. The spatial resolution of each PET scanner was quantified with PET imaging of a Hoffman phantom. For the Aβ PET imaging, the participants were injected with [^18^F]-D3FSP intravenously at 370 MBq (10 mCi ± 10%), rested for 45 min, and prepared for the scanning. [^18^F]-D3FSP Aβ PET/CT imaging was performed 50 min after injection, and the PET acquisition time was 20 min. For the tau PET imaging, the participants were injected with [18F]-flortaucipir intravenously at 370 MBq (10 mCi ± 10%), rested for 75 min, and prepared for imaging. The dynamic acquisition of [18F]-flortaucipir tau PET data was completed 80–100 min after the radiotracer administration.

The PET and MRI images were processed using in-house Matlab algorithms. The PET images were co-registered with their corresponding structural MRI images in SPM12 (Statistical Parametric Mapping). Sixty-eight Freesurfer-defined cortical ROIs obtained from MRI segmentation were used to extract regional FSP and FTP measurements from the co-registered PET images. The FSP SUVR of AD summary cortical regions (posterior cingulate cortex, precuneus, frontal lobe, parietal lobe, and lateral temporal) was obtained by dividing the radiotracer uptake value of AD typical brain regions by that in the brainstem. The FTP SUVR of the AD temporal-metaROI [47] (entorhinal cortex, parahippocampal gyrus, amygdala, inferior temporal and middle temporal brain regions) was used to evaluate cortical tau deposition. For voxel-wise analysis, FSP and FTP SUVR images were intensity-normalized and spatially-normalized at the voxel-wise level in the MNI space in SPM12 (Welcome Department of Imaging Neuroscience, London, UK) as we described previously [8].

### Thresholds of FSP Aβ PET, plasma Aβ_42_/Aβ_40_, and plasma p-Tau_181_

The bimodal distribution of COMPOSITE FSP SUVRs (Supplemental Fig. 1) enables us to use the Gaussian mixed model to estimate two Gaussian distributions of low Aβ and high Aβ to define an unsupervised threshold as COMPOSITE FSP SUVR≥0.76 (Supplemental Fig. 1), which corresponds to a 90% probability of belonging to the high Aβ distribution. The plasma Aβ_42_/Aβ_40_ ratio and plasma p-Tau_181_ did not have clear bimodal distributions. Thus, we defined their thresholds by classifying Aβ PET negative CU individuals and Aβ PET positive CI individuals in the receiver operating characteristic (ROC) curve analysis using the Youden index. The cutoff ≤ 0.0609 for plasma Aβ_42_/Aβ_40_ ratio was defined as the optimal threshold for classifying 154 Aβ- CU participants and 69 Aβ + CI individuals in the ROC curve analysis (Supplemental Figs. 2–3). Similarly, the ROC analysis classified 143 Aβ- CU participants and 67 Aβ + CI participants as the endpoint to define the cutoff ≥ 2.418 for plasma p-Tau_181_ (Supplemental Figs. 4–5). The thresholds of plasma Aβ_42_/Aβ_40_ ratio and plasma p-Tau_181_ divided the whole cohort into 4 different plasma staging profiles: A-/T-, A-/T+, A+/T-, and A+/T + groups.

### Statistical analysis

All the statistical analyses were done using R (v4.3.0, The R Foundation for Statistical Computing). The normal distribution of the data in this study was determined using the Shapiro-Wilk test. We used a two-tailed Mann-Whitney U test and Fisher’s exact test to compare the continuous and categorical characteristics at baseline between different A/T groups, respectively. Data were presented as median (interquartile range, IQR) or No. (%) unless otherwise noted.

Plasma p-Tau_181_, plasma GFAP, and plasma NfL were log_10_ transferred before the following analysis to meet the normal distribution. Generalized linear models (GLM) were used to compare plasma GFAP, COMPOSITE FSP Aβ SUVR, temporal-metaROI FTP SUVR, plasma NfL, rHCV, and temporal-metaROI cortical thickness between different A/T (A: plasma Aβ_42_/Aβ_40_, T: plasma p-Tau_181_) groups, controlling for age, sex, *APOE-ε4*, and diagnosis:

1 $$\begin{gathered} Biomarkers \sim plasma{\text{ }}A/T{\text{ }}profiles{\text{ }} + {\text{ }} \hfill \\\,\,age{\text{ }} + {\text{ }}sex + APOE - \varepsilon 4{\text{ }} + {\text{ }}diagnosis \hfill \\ \end{gathered}$$

Subsequently, we used GLM models to explore the association of plasma GFAP with plasma Aβ_42_/Aβ_40_ and plasma p-Tau_181_, including the same covariates above:

2 $$\begin{gathered} Plasma{\text{ }}GFAP \sim plasma\,A{\beta _{42}}/A{\beta _{40}} \times \hfill \\\,\,\,\,\,\,\,plasma{\text{ }}p - Ta{u_{181}} + {\text{ }}covariates \hfill \\ \end{gathered}$$

Besides, we investigated the association of plasma GFAP with plasma NfL, FSP SUVR, FTP SUVR, rHCV, and temporal-metaROI cortical thickness, including the same covariates above as well as without (model 3) and with (model 4), including plasma Aβ_42_/Aβ_40_ and plasma p-Tau_181_ in the model:

3 $$Biomarkers \sim plasma{\text{ }}GFAP{\text{ }} + {\text{ }}covariates$$

4 $$\begin{gathered} Biomarkers \sim plasma{\text{ }}GFAP{\text{ }} + {\text{ }}plasma\,A{\beta _{42}}/A{\beta _{40}} + {\text{ }} \hfill \\\,\,\,\,\,\,\,\,\,\,\,\,\,\,\,\,\,\,\,\,\,\,\,\,\,plasma{\text{ }}p - Ta{u_{181}} + {\text{ }}covariates \hfill \\ \end{gathered}$$

Furthermore, we investigated the independent and synergistic effect of plasma Aβ_42_/Aβ_40_, plasma p-Tau_181_, and plasma GFAP with plasma NfL, FSP SUVR, FTP SUVR, rHCV, and temporal-metaROI cortical thickness, including the same covariates above:

5 $$\begin{gathered} Biomarkers \sim plasmaA{\beta _{42}}/A{\beta _{40}} \times plasma{\text{ }}p - \hfill \\ \,\,\,\,\,\,\,\,\,\,\,Ta{u_{181}} \times plasma{\text{ }}GFAP{\text{ }} + {\text{ }}covariates \hfill \\ \end{gathered}$$

Notably, all the statistic in these models were obtained based on the continuous variables in order to avoid any influences from the pre-defined thresholds. To better illustrate the association of plasma GFAP with plasma Aβ_42_/Aβ_40_ and plasma p-Tau_181_, as well as how plasma GFAP modulates the association of plasma Aβ_42_/Aβ_40_ and plasma p-Tau_181_ with FSP SUVR, FTP SUVR, plasma NfL, rHCV, and temporal-metaROI cortical thickness, we plotted the association in the analysis of models (2) and (5) above in individuals with high (> Median) and low (< Median) plasma Aβ_42_/Aβ_40_, plasma p-Tau_181_, and plasma GFAP separately. The *Pearson* correlation test calculated the correlation coefficient *R* between outcome and predictor in individuals with high (> Median) and low (< Median) plasma Aβ_42_/Aβ_40_, plasma p-Tau_181_, and plasma GFAP.

Subsequently, we investigated the voxel-wise association between plasma Aβ_42_/Aβ_40_ and FSP Aβ PET images in all the participants with FSP Aβ PET images, participants with high (> Median) and low (< Median) plasma p-Tau_181_ concentrations separately, controlling for the same covariates above. Furthermore, we studied the voxel-wise association of FTP tau PET images with plasma p-Tau_181_ in all the individuals, individuals with high (> Median) and low (< Median) plasma GFAP concentrations separately, including the same covariates above. The voxel-wise associations between plasma Aβ_42_/Aβ_40_ and FSP Aβ PET images and between plasma p-Tau_181_ and FTP tau PET images were presented using an uncorrected voxel threshold of *p* < 0.001, and the T-maps were converted into R-maps using the CAT12 toolbox (www.neuro.uni-jena.de/cat/) and displayed with family-wise error (FWE) corrected *p* < 0.05.

To evaluate how plasma GFAP modulates the voxel-wise association of cortical thickness images with plasma Aβ_42_/Aβ_40_ and plasma p-Tau_181_, we resampled the cortical thickness images, mapped them to the fsaverage space and smoothed with a kernel size of 10 mm full-width at half maximum. We finally determined the vertex-wise association of the cortical thickness images with plasma Aβ_42_/Aβ_40_ and plasma p-Tau_181_, controlling for the same covariates above. We did the vertex-wise analysis in all the participants with MRI data, participants with high (> Median) and low (< Median) plasma GFAP concentrations separately. The vertex-wise association between cortical thickness images and plasma p-Tau_181_ was presented using an uncorrected voxel threshold of *p* < 0.001 (Monte Carlo simulation corrected for multiple comparisons) with FWE corrected *p* < 0.05 at the cluster level, and the statistical results were overlaid onto inflated cortical surfaces. For plasma Aβ_42_/Aβ_40_, we only showed the results of an uncorrected voxel threshold of *p* < 0.001 because no significant cluster survived after correction.

## Results

### Demographics of participants

The demographic characteristics of participants at baseline are summarized in Table 1. At baseline, A+/T + individuals had older ages, higher percentages of *APOE-ε4* carriers, and lower MoCa and MMSE scores than A-/T- individuals. The A+/T + group also had higher percentages of *APOE-ε4* carriers and lower MoCa and MMSE scores than the A-/T + and A+/T- groups. Moreover, 301, 195, and 70 individuals had concurrent MRI, ^18^F-D3-FSP Aβ PET, and FTP tau PET scans.

**Table 1:** Demographics of participants included in this study

	A-/T-	A-/T+	A+/T-	A + T+
**No.**,** %**	206, 43.8%	60, 12.8%	121, 25.7%	83, 17.7%
**CI (No.**,** %)**	27, 22.1%	23, 38.3%	35, 28.9%	59, 71.1%
**Age**,** years**	65 (8.7,56–86)	^a^69 (11.2,55–86)	^a^67 (8.8,55–85)	^a, b^72 (9.9,56–89)
**APOE-ε4 (No.**,** %)**	35, 17.0%	^a^18, 30.0%	27, 22.3%	^a, b, c^42, 50.6%
**Female (No.**,** %)**	142, 68.9%	33, 55.0%	84, 69.4%	46, 55.4%
**MoCa**	26.0 (4.0)	^a^25.0 (5.25)	26.0 (5.0)	^a, b, c^16.0 (12.5)
**MMSE**	29.0 (3.0)	^a^28.0 (4.0)	^a^280 (3.0.)	^a, b, c^24.0 (10.0.)
**Participants with MRI image data (** *n* ** = 301)**				
**No.**,** %**	122, 40.5%	43, 14.3%	74, 24.6%	62, 20.6%
**CI (No.**,** %)**	18, 14.8%	14, 32.6%	22, 29.7%	42, 67.7%
**Age**,** years**	66 (7.4,56–81)	68 (10.7,55–86)	67 (8.3,55–83)	^a, b^72 (10.1,56–89)
**APOE-ε4 (No.**,** %)**	27, 22.1%	10, 23.3%	15, 20.3%	^a, b, c^33, 53.2%
**Female (No.**,** %)**	81, 66.4%	24, 55.8%	52, 70.3%	36, 58.1%
**MoCa**	26.0 (3.75)	^a^25. (5.5)	26.0 (5.0)	^a, b, c^19.0 (12.75)
**MMSE**	29.0 (3.0.)	^a^28.0 (4.0)	^a^28.0 (3.0)	^a, b, c^24.0 (10.75)
**Participants with Aβ PET image data (** *n* ** = 195)**				
**No.**,** %**	78, 40.0%	30, 15.4%	41, 21.0%	46, 23.6%
**CI (No.**,** %)**	13,16.7%	12, 40%	15, 36.6%	32, 69.6%
**Age**,** years**	66 (8.6,55–89)	67 (7.4,55–81)	^a, b^69 (9.1,58–89)	^a, b^73 (12.7,58–85)
**APOE-ε4 (No.**,** %)**	18, 23.1%	7, 23.3%	^b^8, 19.5%	^a, b, c^25, 54.3%
**Female (No.**,** %)**	48, 61.5%	16, 53.3%	28, 68.3%	25, 54.3%
**MoCa**	26.0 (3.0)	^c^25.0 (6.0)	26.0 (4.0.)	^a, b, c^20.0. (12.0)
**MMSE**	29.0 (3.0)	^a^27.0 (4.75)	^a^27.0 (30)	^a, b, c^24.0 (9.75)
**Participants with tau PET image data (** *n* ** = 70)**				
**No.**,** %**	16, 22.9%	10, 14.3%	18, 25.7%	26, 37.1%
**CI (No.**,** %)**	4, 25.0%	4, 40.0%	5, 27.8%	16, 61.5%
**Age**,** years**	64(7.1.7,57–77)	65(8.2,58–77)	66(8.7,55–83)	^a^71(12.7,56–89)
**APOE-ε4 (No.**,** %)**	9, 56.3%	4, 40.0%	6, 33.3%	17, 65.4%
**Female (No.**,** %)**	8, 50.0%	5, 50.0%	12, 66.7%	15, 57.8%
**MoCa**	28.0 (2.5)	25.5 (7.0)	^a^24.5 (4.0)	^a, b, c^18.5 (11.75)
**MMSE**	29.0 (1.25)	27.0 (2.5)	^b^27.5 (2.75)	^a, b, c^24 (9.75)

Note: ^*a, b,c*^*indicate significantly different from A-/T-*,* A+/T-*,* and A-/T + groups respectively*

### Comparisons of plasma biomarkers and neuroimages among different A/T stages

**Fig. 1:**
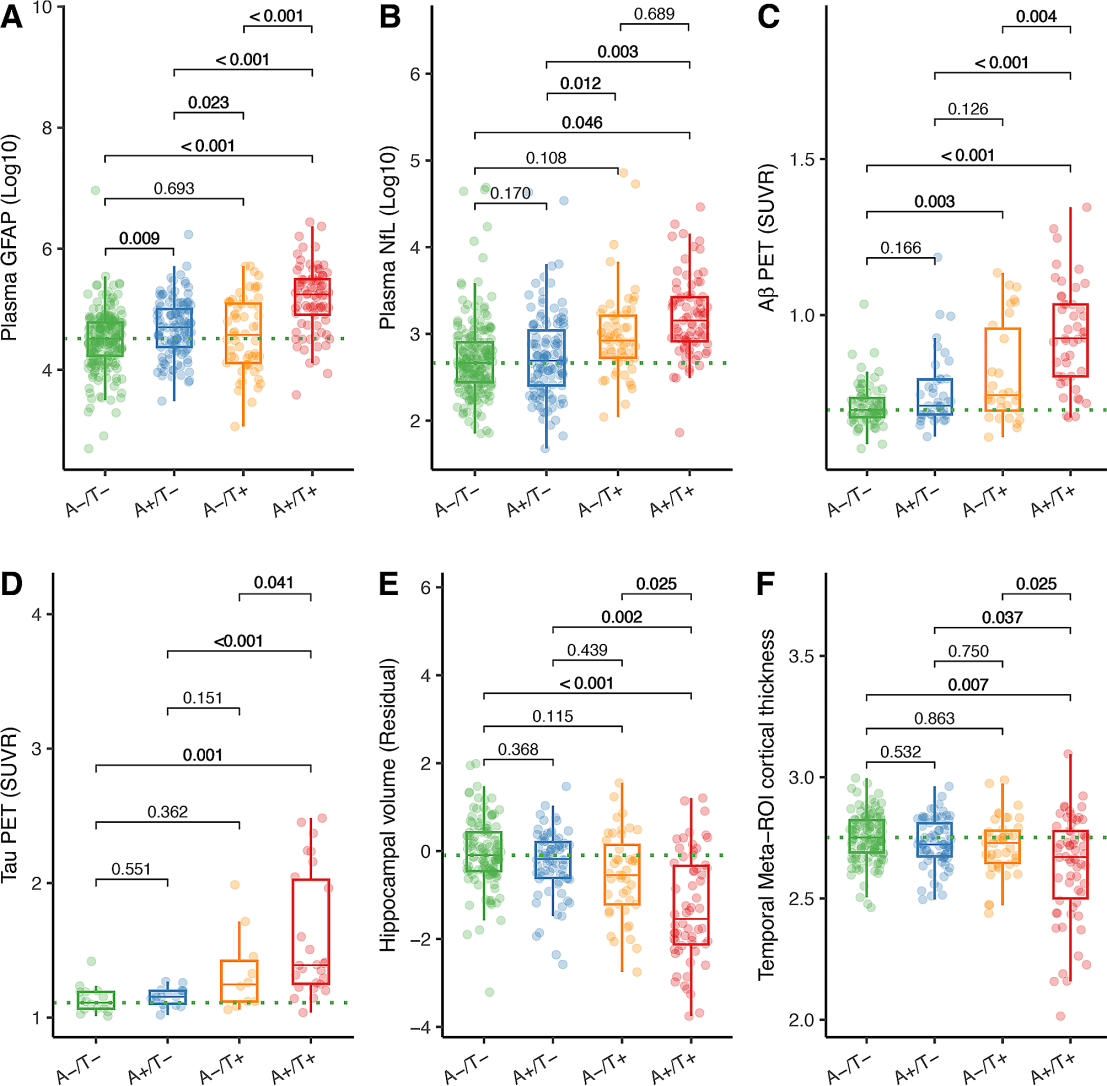
Comparisons of plasma biomarkers and neuroimaging between different A/T profiles. Comparisons of **(A)** plasma GFAP, **(B)** plasma NfL, **(C)** Aβ PET, **(D)** tau PET, **(F)** residual hippocampal volume (rHCV), and **(G)** temporal-MetaROI cortical thickness between A-/T-, A+/T-, A-/T+, and A+/T + groups. Plasma GFAP and plasma NfL were log_10_ transferred before they were used in the general linear models. For the boxplots, each point represents an individual, and the dashed lines represent the median values of the A-/T- group. The median (horizontal bar), interquartile range (IQR, hinges), and 1.5 × IQR (whiskers) were presented in boxplots as well. The *p* values of the comparisons were shown at the top, adjusting for age, sex, *APOE*-ε4 status, and diagnosis

Regarding the comparisons of different A/T profiles defined by plasma Aβ_42_/Aβ_40_ (A) and plasma p-Tau_181_ (T), we found that A + T + individuals showed higher plasma GFAP, COMPOSITE Aβ PET SUVR, temporal-metaROI FTP SUVR, and lower rHCV, and temporal-metaROI cortical thickness than A-/T-, A-/T+, and A+/T- groups (Fig. 1A, C-F). Besides, A + T + individuals only had higher plasma NfL than A-/T-group (Fig. 1B). The *Supplemental Material* provided more details on the comparisons between the A+/T + group and other groups.

Furthermore, A-/T + individuals had higher plasma NfL concentrations than A+/T- group (standardized β (*β*_*std*_) = 0.316[95% confidence interval (ci), 0.069, 0.563], Fig. 1B), and higher COMPOSITE Aβ PET SUVR than A-/T- group (*β*_*std*_ = 0.462[95% ci, 0.153, 0.770], Fig. 1C). In addition, A+/T- individuals showed higher plasma GFAP concentrations than A-/T- group (*β*_*std*_ = 0.253[95% ci, 0.063, 0.443], Fig. 1A) and A-/T+ (*β*_*std*_ = 0.303[95% ci, 0.041, 0.565], Fig. 1A) group.

### Association of plasma Aβ_42_/Aβ_40_, p-Tau_181_, GFAP and NfL

**Fig. 2:**
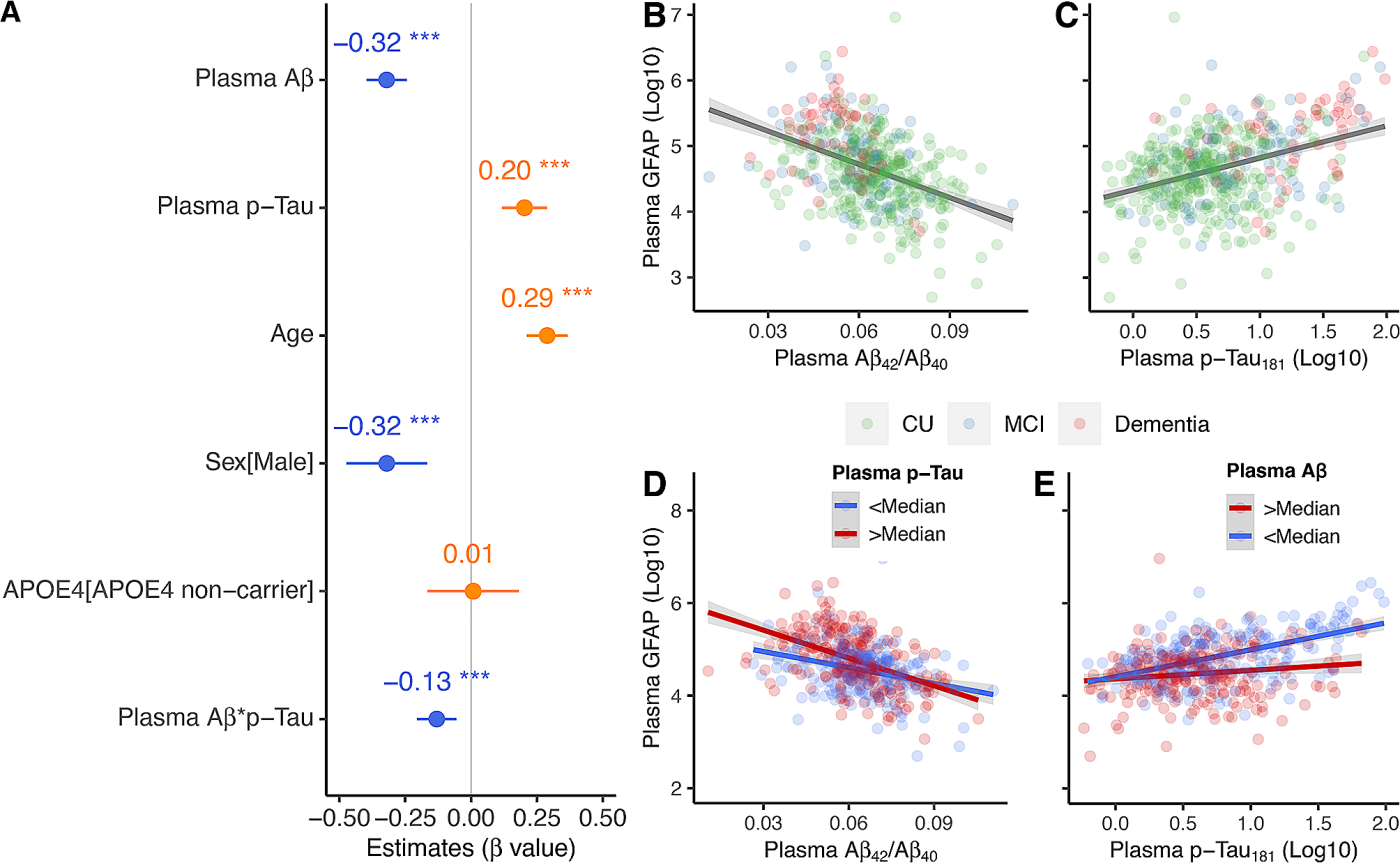
Association of plasma Aβ_42_ /Aβ_40_ and plasma p-Tau _181_ with plasma GFAP. Association of plasma Aβ_42_/Aβ_40_ and plasma p-Tau_181_ with plasma GFAP **(A-C)**. For better illustration, the association of plasma GFAP with plasma Aβ_42_/Aβ_40_ and plasma p-Tau_181_ were presented in different subgroups (< Median and > Median) of **(D)** plasma p-Tau_181_ and **(E)** plasma Aβ_42_/Aβ_40_, but the interactive effect on plasma GFAP of plasma Aβ_42_/Aβ_40_ and plasma p-Tau_181_ were conducted using continuous variables. Plasma p-Tau_181_ and plasma GFAP were log_10_ transferred before they were used in the general linear models

For the astrocyte reactivity measured by plasma GFAP, we found lower plasma Aβ_42_/Aβ_40_ (Fig. 2B, *β*_*std*_ = -0.320[95% ci, -0.397, -0.244], *p* < 0.001), higher plasma p-Tau_181_ (Fig. 2C, *β*_*std*_ = 0.203[95% ci, 0.117, 0.288], *p* < 0.001), older ages (*β*_*std*_ = 0.289[95% ci, 0.211, 0.367], *p* < 0.001), and females (*β*_*std*_ = 0.320[95% ci, 0.167, 0.473], *p* < 0.001) were related to higher plasma GFAP levels. Lower plasma Aβ_42_/Aβ_40_ and higher plasma p-Tau_181_ concentrations showed significant interaction (Fig. 2A, *β*_*std*_ = -0.130[95% ci, -0.205, -0.055], *p* < 0.001) with higher plasma GFAP levels. Specifically, the negative association between plasma Aβ_42_/Aβ_40_ and plasma GFAP was marginally stronger (Fig. 2D, *β*_*std*_ = -0.145[95% ci, -0.297, 0.007], *p* = 0.061) among individuals with high (> median) plasma p-Tau_181_ concentrations than those with low plasma p-Tau_181_ concentrations. The positive association between plasma p-Tau_181_ and plasma GFAP was more robust (Fig. 2E, *β*_*std*_ = 0.281[95% ci, 0.123, 0.439], *p* < 0.001) in individuals with low (< median) plasma Aβ_42_/Aβ_40_ than that in individuals with high (> median) plasma Aβ_42_/Aβ_40_.

We further determined the association of plasma Aβ_42_/Aβ_40_, plasma p-Tau_181_, and plasma GFAP with plasma NfL. No significant interaction was found between plasma Aβ_42_/Aβ_40_, plasma p-Tau_181_, and plasma GFAP in predicting the levels of plasma NfL, although higher plasma p-Tau_181_ and plasma GFAP concentrations, older ages, and males were associated with higher plasma NfL levels (Supplemental Fig. 6).

### Association of plasma Aβ_42_/Aβ_40_, p-Tau_181_, and GFAP with Aβ plaques and tau tangles

Regarding the association of plasma GFAP, plasma Aβ_42_/Aβ_40_ and plasma p-Tau_181_ with Aβ PET and tau PET, we found that plasma GFAP was related to higher Aβ PET regardless of including plasma Aβ_42_/Aβ_40_ and plasma p-Tau_181_ as the covariates (Supplemental Fig. 7, before including covariates: β_std_ = 0.300[95% ci, 0.181, 0.419], *p* < 0.001; after including covariates: β_std_ = 0.152[95% ci, 0.032, 0.273], *p* = 0.022) and tau PET (β_std_ = 0.470[95% ci, 0.288, 0.651], *p* < 0.001; β_std_ = 0.335[95% ci, 0.139, 0.461], *p* < 0.001).

**Fig. 3:**
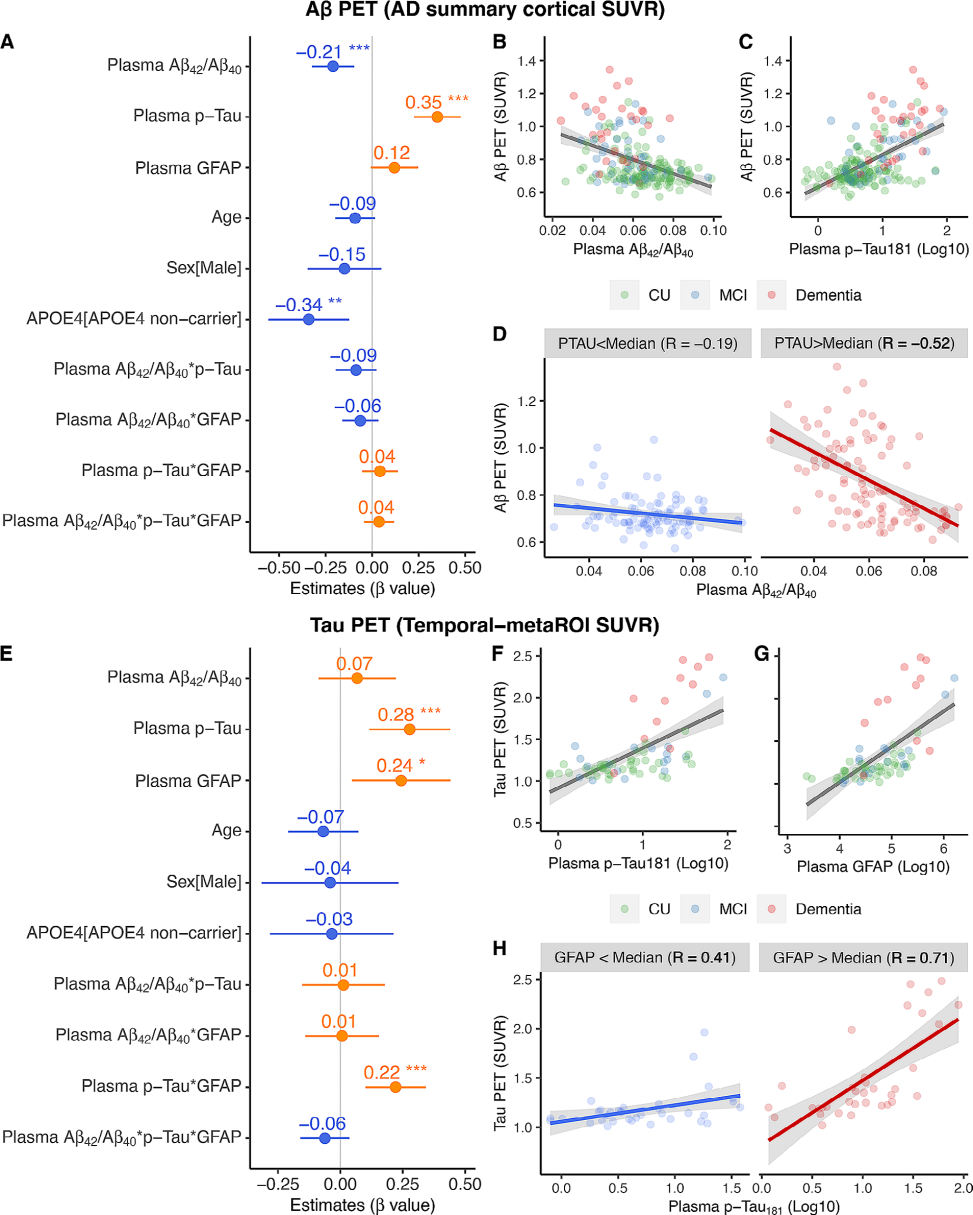
Association of plasma Aβ_42_ /Aβ_40_, plasma p-Tau_181_, and plasma GFAP with Aβ PET and tau PET. Association of plasma Aβ_42_/Aβ_40_, plasma p-Tau_181_, and plasma GFAP with **(A-C)** Aβ PET and **(E-G)** tau PET. Notably, plasma p-Tau_181_ and plasma GFAP were log_10_ transferred before they were used in the general linear models. Notably, the interactive effects on Aβ PET and tau PET of plasma Aβ_42_/Aβ_40_, plasma p-Tau_181_, and plasma GFAP were conducted using continuous variables. For better illustration, **(D)** the association between Aβ PET and plasma Aβ_42_/Aβ_40_ was presented in different subgroups (< Median and > Median) of plasma p-Tau_181_, and **(H)** the association between tau PET and plasma p-Tau_181_ was presented in different subgroups (< Median and > Median) of plasma GFAP.

Subsequently, we investigated the independent and synergistic predictive effect of plasma Aβ_42_/Aβ_40_, p-Tau_181_, and GFAP at cortical Aβ plaques and tau tangles. Lower plasma Aβ_42_/Aβ_40_ (*β*_*std*_ = -0.210[95% ci, -0.324, -0.097], *p* < 0.001), higher plasma p-Tau_181_ concentrations (*β*_*std*_ = 0.351[95% ci, 0.226, 0.475], *p* < 0.001), and *APOE-ε4* carriers (*β*_*std*_ = 0.341[95% ci, 0.123, 0.558], *p* = 0.002) were associated with higher Aβ PET SUVR (Fig. 3A-C), whereas the relation between plasma GFAP and Aβ PET SUVR became marginal (*β*_*std*_ = 0.120[95% ci, -0.007, 0.247], *p* = 0.064). Moreover, the negative association between plasma Aβ_42_/Aβ_40_ and Aβ PET SUVR was stronger in individuals with high (> median) plasma p-Tau_181_ concentrations (*R* = -0.52[95% ci, -0.651, -0.358], *p* < 0.001) than those with low (< median) plasma p-Tau_181_ concentrations (*R* = -0.19[95% ci, -0.371, 0.014], *p* = 0.069) (Fig. 3D). In contrast, higher plasma p-Tau_181_ (Fig. 3F, *β*_*std*_ = 0.278[95% ci, 0.117, 0.439], *p* < 0.001) and plasma GFAP (Fig. 3G, *β*_*std*_ = 0.244[95% ci, 0.047, 0.441], *p* = 0.015) concentrations but not plasma Aβ_42_/Aβ_40_ were associated with higher temporal-metaROI tau PET SUVR. Notably, plasma p-Tau_181_ and plasma GFAP showed significant interactive relation (Fig. 3E, *β*_*std*_ = 0.221[95% ci, 0.101, 0.342], *p* < 0.001) with higher tau PET SUVR. Specifically, the positive association between plasma p-Tau_181_ and tau PET SUVR was more robust (Fig. 3H) in individuals with high (> median) plasma GFAP concentrations (*R* = 0.71[95% ci, 0.488, 0.842], *p* < 0.001) than in individuals with low (< median) plasma GFAP concentrations (*R* = 0.41[95% ci, 0.088, 0.653], *p* = 0.015).

**Fig. 4:**
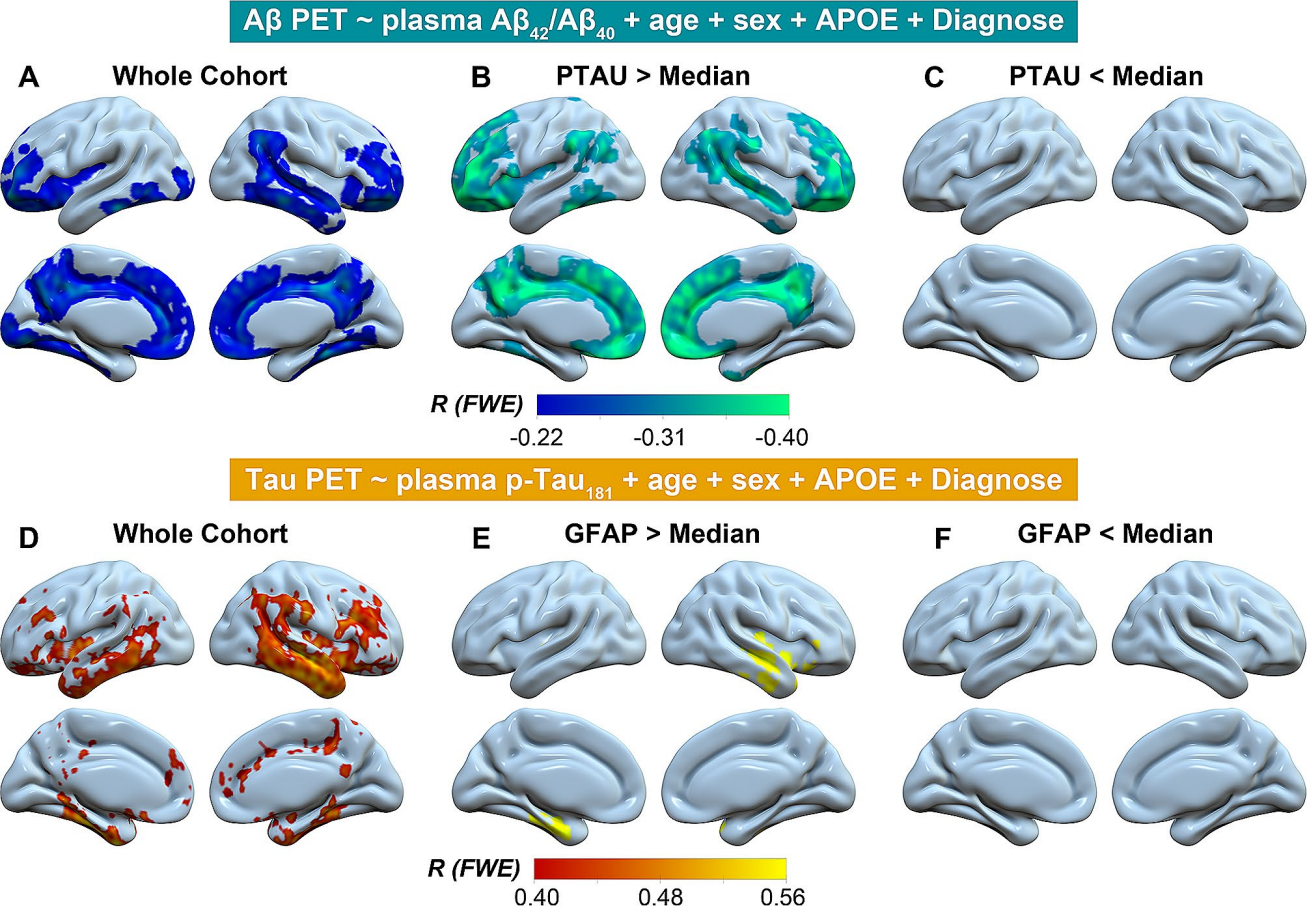
Voxel-wise association of plasma Aβ_42_/Aβ_40_, plasma p-Tau_181_with Aβ PET and tau PET. Voxel-wise association of plasma Aβ_42_/Aβ_40_ and plasma p-Tau_181_ with **(A-C)** Aβ PET and **(D-F) **tau PET in the whole cohort, individuals with plasma p-Tau_181_ or plasma GFAP > Median and < Median. Notably, Plasma p-Tau_181_ and plasma GFAP were log_10_ transferred before they were used in the general linear models

The voxel-wise analysis was consistent with the ROI analysis, where we found plasma Aβ_42_/Aβ_40_ and Aβ PET SUVR showed significant negative association in the whole cohort and individuals with high (> median) plasma p-Tau_181_ concentrations but not in individuals with low (< median) plasma p-Tau_181_ concentrations (Fig. 4A-C). The voxel-wise analysis between plasma p-Tau_181_ and FTP tau PET images replicated the ROI analysis findings (Fig. 4D-F).

### Association of plasma Aβ_42_/Aβ_40_, p-Tau_181_, and GFAP with hippocampal atrophy and cortical thinning

Regarding the hippocampal atrophy and AD-signature cortical thinning, higher plasma GFAP concentrations were associated with more hippocampal atrophy (β_std_ = -0.146[95% ci, -0.246, -0.047], *p* = 0.004) before controlling for plasma Aβ_42_/Aβ_40_ and plasma p-Tau_181_, whereas the relation disappeared (β_std_ = -0.066[95% ci, -0.171, 0.039], *p* = 0.218) after including them as the covariates in the model (Supplemental Fig. 8A-B). In contrast, elevated plasma GFAP levels were correlated with more temporal-metaROI cortical thinning regardless of controlling for plasma Aβ_42_/Aβ_40_ and plasma p-Tau_181_ (Supplemental Fig. 8C-D, before including covariates: β_std_ = -0.210[95% ci, -0.322, -0.098], *p* < 0.001; after including covariates: β_std_ = -0.176[95% ci, -0.297, -0.054], *p* = 0.011).

**Fig. 5:**
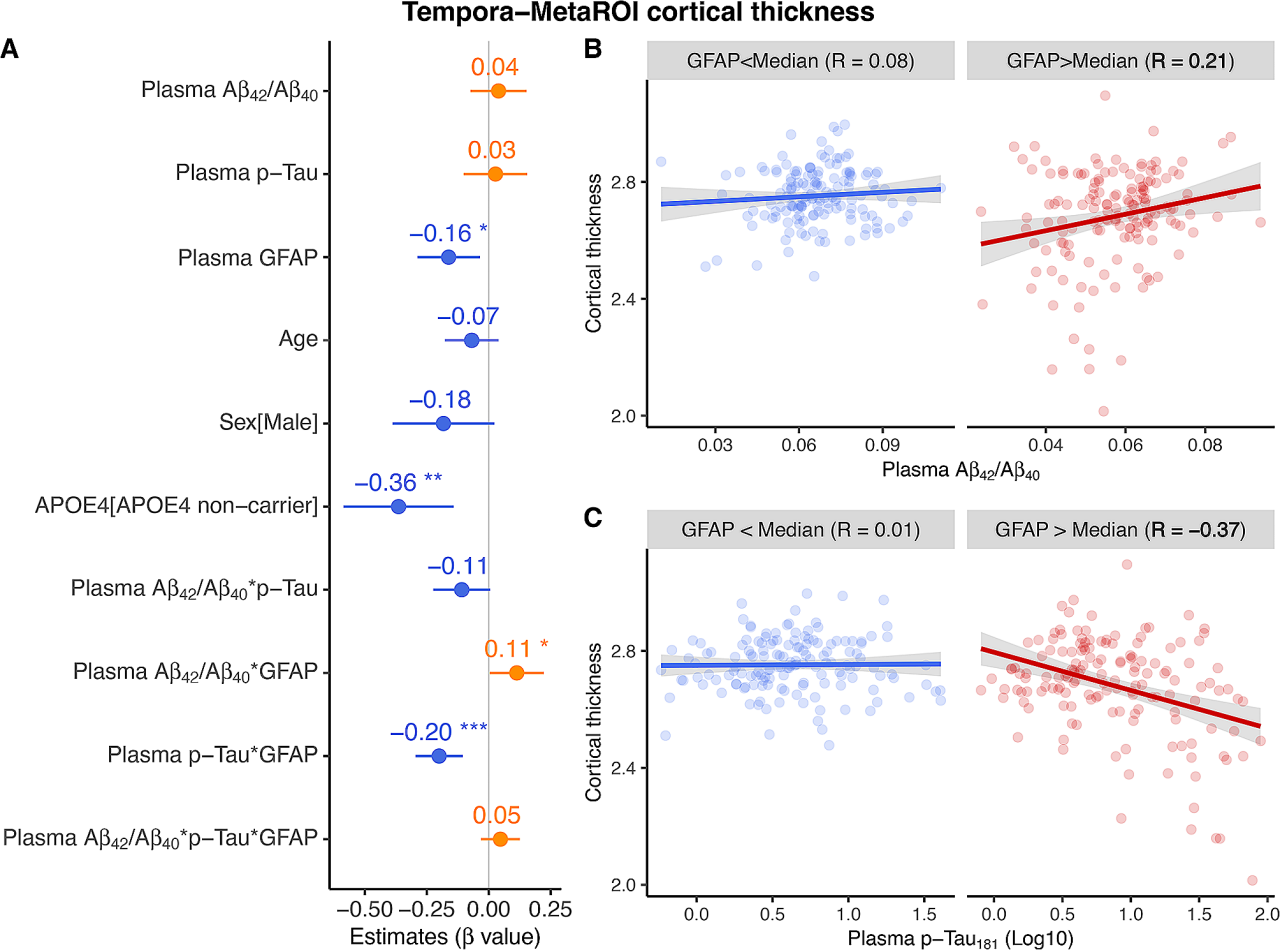
Association of plasma Aβ_42_/Aβ_40_, plasma p-Tau_181_, and plasma GFAP with cortical thinning. Association of plasma Aβ_42_/Aβ_40_, plasma p-Tau_181_, and plasma GFAP with **(A)** temporal-metaROI cortical thickness. Notably, Plasma p-Tau_181_ and plasma GFAP were log_10_ transferred before they were used in the general linear models. Notably, the interactive effect of plasma Aβ_42_/Aβ_40_ and plasma p-Tau_181_ with plasma GFAP on temporal-metaROI cortical thinning were conducted using continuous variables. For better illustration, the association of temporal-metaROI cortical thickness with **(B)** plasma Aβ_42_/Aβ_40_ and **(C) **plasma p-Tau_181_ was presented in different subgroups (< Median and > Median) of plasma GFAP

**Fig. 6:**
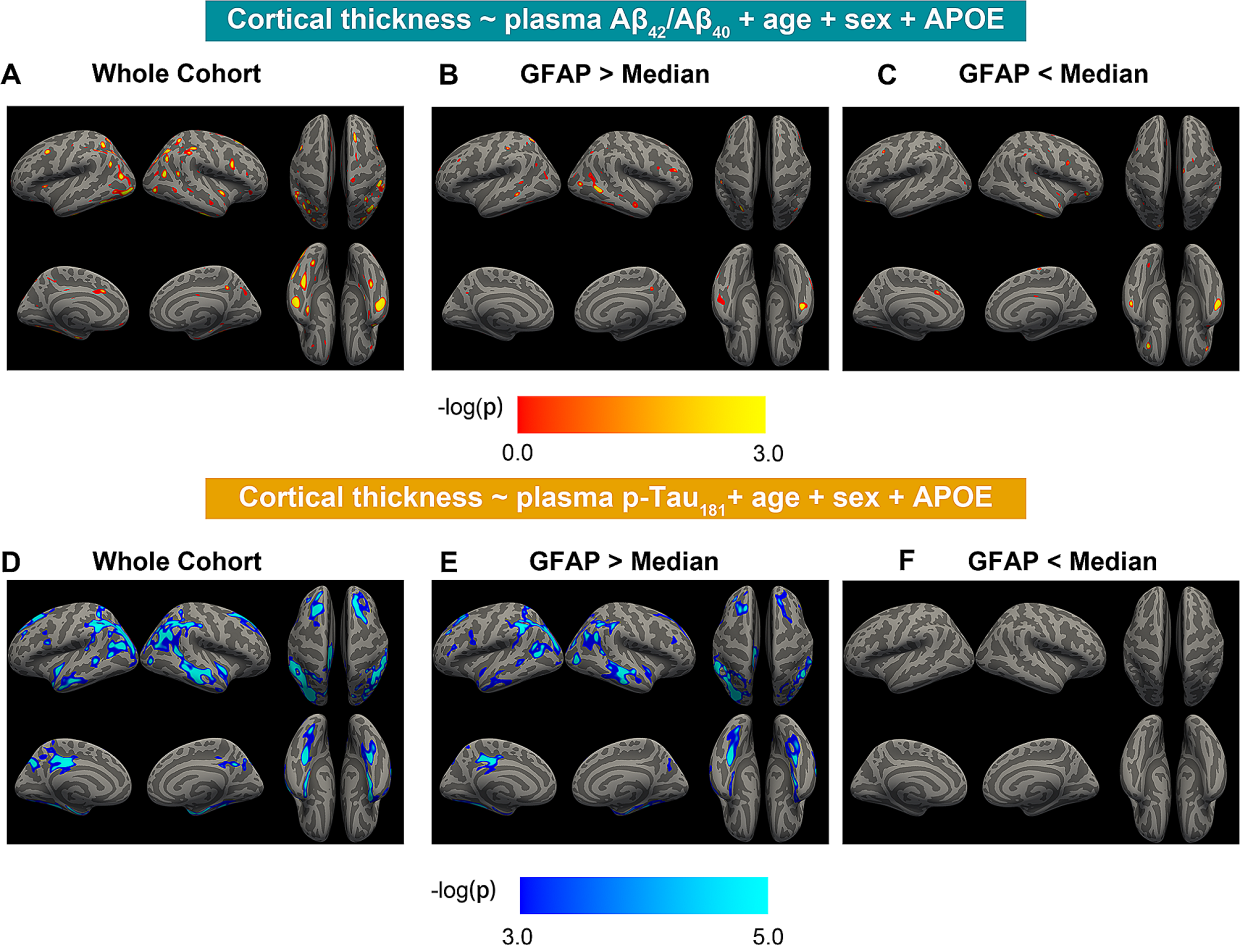
Vertex-wise association of plasma Aβ_42_/Aβ_40_and plasma p-Tau_181_with cortical thickness images. Vertex-wise association of cortical thickness images with **(A-C)** plasma Aβ_42_/Aβ_40_ and **(D-F)** plasma p-Tau_181_ in the whole cohort, individuals with plasma p-Tau_181_ or plasma GFAP > Median and < Median. The plasma Aβ_42_/Aβ_40_ results were shown with an uncorrected voxel threshold of *p* < 0.001. For plasma p-Tau_181_, the results were presented using an uncorrected voxel threshold of *p* < 0.001 with family-wise error corrected *p* < 0.05 at the cluster level, and the statistical results were overlaid onto inflated cortical surfaces. Notably, Plasma p-Tau_181_ and plasma GFAP were log_10_ transferred before they were used in the general linear models

In models aiming to investigate the independent and interactive effect of plasma GFAP, plasma Aβ_42_/Aβ_40_, and plasma p-Tau_181_ on hippocampal atrophy and AD-signature cortical thinning, we found higher plasma GFAP concentrations (β_std_ = -0.162[95% ci, -0.288, -0.035], *p* = 0.012) and *APOE-ε4* non-carriers (β_std_ = -0.363[95% ci, -0.586, -0.141], *p* = 0.001) were related to more shrinking in temporal-metaROI cortical thickness (Fig. 5A). Additionally, plasma GFAP showed significant interaction with plasma Aβ_42_/Aβ_40_ (β_std_ = 0.113[95% ci, 0.004, 0.223], *p* = 0.042) and plasma p-Tau_181_ (β_std_ = -0.200[95% ci, -0.296, -0.104], *p* < 0.001) at predicting temporal-metaROI cortical thinning. To be more specific, the associations of plasma Aβ_42_/Aβ_40_ and plasma p-Tau_181_ with temporal-metaROI cortical thickness were more robust in individuals with high (> median) plasma GFAP concentrations (plasma Aβ_42_/Aβ_40_: *R* = 0.21[95% ci, 0.051, 0.357], *p* = 0.010; plasma p-Tau_181_: *R* = -0.37[95% ci, -0.497, -0.219], *p* < 0.001) than in individuals with low (< median) plasma GFAP concentrations (plasma Aβ_42_/Aβ_40_: *R* = 0.08[95% ci, -0.081, 0.238], *p* = 0.326; plasma p-Tau_181_: *R* = 0.01[95% ci, -0.149, 0.171], *p* = 0.893) (Fig. 5B-C). The vertex-wise analysis of cortical thickness images with plasma Aβ_42_/Aβ_40_ and plasma p-Tau_181_ substantially replicated the ROI analysis findings (Fig. 6A-F). In contrast, we did not find interaction in any biomarkers on hippocampal atrophy, although we observed that lower plasma Aβ_42_/Aβ_40_ (*β*_*std*_ = 0.117[95% ci, 0.015, 0.218], *p* = 0.024), higher plasma p-Tau_181_ concentrations (*β*_*std*_ = -0.190[95% ci, -0.306, -0.075], *p* = 0.001), and older ages (*β*_*std*_ = -0.274[95% ci, -0.371, -0.176], *p* < 0.001) but not plasma GFAP concentrations were related to more decreases in rHCV (Supplemental Fig. 9).

## Discussion

In this study, we established the thresholds for plasma Aβ_42_/Aβ_40_ and plasma p-Tau_181_ and demonstrated that individuals positive for these biomarkers (A+/T+) exhibited the most significant alterations in plasma GFAP, Aβ PET, tau PET, plasma NfL, hippocampal volume, and AD-signature cortical thickness within a Chinese community-based aging cohort. Higher astrocyte reactivity, as measured by plasma GFAP, was strongly linked with tau tangle aggregation and cortical thickness thinning in AD. Importantly, the thresholds of plasma Aβ_42_/Aβ_40_ and plasma p-Tau_181_ reported in this study are significant references for detecting AD in the older Chinese Han community population. These findings provide novel insights into the association among Aβ, tau, astrocyte reactivity, and neurodegeneration in AD.

Currently, there are no definitive thresholds for AD core plasma biomarkers in the Chinese Han community population. Previous clinical-based studies [50–52] have reported the potential of plasma biomarkers to identify CI individuals or predict cognitive decline in the Chinese Han population. In the present study, we recruited a large Chinese Han population from the community in South China to define the cutoffs for plasma Aβ_42_/Aβ_40_ and plasma p-Tau_181_ by comparing a large dataset of Aβ PET negative CU individuals and Aβ PET positive CI individuals. Consistent with the previous studies [36–40], one of the key findings in the present study was that plasma A+/T + individuals, defined by plasma Aβ_42_/Aβ_40_ and plasma p-Tau_181_, showed the highest levels of astrocyte reactivity, Aβ plaque, tau tangle, axonal degeneration, hippocampal atrophy, and cortical thinning. This supports the feasibility of using plasma Aβ_42_/Aβ_40_ and plasma p-Tau_181_ to detect AD in the Chinese community older population. Compared to the plasma A-/T- group, plasma A+/T- individuals have higher plasma GFAP concentrations, whereas plasma A-/T + individuals had more Aβ plaques. This indicates that plasma A+/T- individuals may exhibit astrocyte reactivity, whereas plasma A-/T + individuals may have early alternations in Aβ plaques rather than belong to the primary age-related tauopathy (PART) [53]. Consistent with our findings, one recent study [54] did not find significant increases in plasma p-Tau concentrations in individuals with PART defined by autopsy data. The cutoff of 0.0609 for the plasma Aβ_42_/Aβ_40_ ratio defined in the present study was comparable with the previously reported value with the autopsy data as the standard based on the European population [55]. Notably, the cutoff of 2.418 for plasma p-Tau181 was much more lenient than the value of 3.962 defined for the European population [55]. The different types of pre-analytical procedure [56], analytical [57], and real-world community studies [58] may explain the discrepancy. Future studies with larger and independent datasets in the Chinese Han community population are essential to validate the thresholds of plasma Aβ_42_/Aβ_40_ and plasma p-Tau_181_ defined in the present study.

The regression findings were also consistent with the dichotomous analyses. Specifically, lower plasma Aβ_42_/Aβ_40_ was correlated with higher plasma GFAP concentrations and Aβ plaques, while higher plasma p-Tau_181_ was related to higher plasma GFAP concentration, Aβ plaque, tau tangle, plasma NfL concentration, and more hippocampal atrophy. This further supports that plasma p-Tau_181_ may be more closely linked to AD pathological changes than plasma Aβ_42_/Aβ_40_. In line with our findings, the BIOFINDER group [59] reported that plasma Aβ_42_/Aβ_40_ predicted longitudinal Aβ accumulation, while higher plasma p-Tau_217_ concentrations were associated with longitudinal tau accumulation. Besides, the Mayo group [36] noted that both plasma Aβ_42_/Aβ_40_ and plasma p-Tau_181_ may be related to cortical Aβ burden, with plasma p-Tau_181_ showing the most improved discrimination of cortical tau tangles. Furthermore, lower plasma Aβ_42_/Aβ_40_ and higher plasma p-Tau_181_ had a marginal synergistic effect in predicting higher Aβ plaques, where individuals with high (> Median) plasma p-Tau_181_ concentrations showed a stronger association with Aβ PET than those with low (< Median) concentrations. Together, these findings support the recommendation of staging AD biologically using both plasma Aβ_42_/Aβ_40_ and plasma p-Tau_181_ biomarkers rather than plasma Aβ_42_/Aβ_40_ alone, as proposed by the NIA-AA revised clinical criteria for AD at AAIC 2023.

Previous literature [30, 31, 41–43] suggested plasma GFAP may be an early AD biomarker. One very recent study [29] demonstrated that plasma GFAP concentrations may modulate the relation between Aβ and tau in preclinical AD. Pelkmans and colleagues [44] also found that plasma GFAP may mediate the association between soluble and insoluble Aβ pathology in CU individuals. In the present study, we further demonstrated that the astrocyte reactivity measured by plasma GFAP significantly influences the association between plasma Aβ_42_/Aβ_40_ and plasma p-Tau_181_ and their relations with the downstream events of AD, including AD-signature cortical tau aggregation and cortical thinning. Specifically, lower plasma Aβ_42_/Aβ_40_ was correlated with higher plasma p-Tau_181_ concentrations and lower temporal-metaROI cortical thickness only in the presence of high astrocyte reactivity reflected by plasma GFAP. The plasma p-Tau_181_-related higher cortical tau tangle aggregation and lower temporal-metaROI cortical thinning were more robust among individuals with high plasma GFAP concentrations than those with low plasma GFAP concentrations. Both higher plasma p-Tau_181_ and plasma GFAP concentrations were related to higher plasma NfL concentrations, but no interaction was observed. Notably, higher plasma GFAP concentrations were unrelated to more cortical Aβ plaque accumulation or hippocampal atrophy, nor did they influence the association of plasma Aβ_42_/Aβ_40_ and plasma p-Tau_181_ with cortical Aβ plaque accumulation or hippocampal atrophy. Together, these results suggest that the inflammation (I) biomarker plasma GFAP may play a cortical role in tau aggregation and cortical thinning in addition to plasma A/T biomarkers.

Consistent with previous findings [2, 60], we found a strong age effect in astrocyte activity measured by plasma GFAP, axonal degeneration measured by plasma NfL, and hippocampal atrophy. In contrast, no significant association with age was found in AD-signature cortical Aβ plaque accumulation, tau tangle aggregation, and cortical thinning. These findings suggest that Aβ PET, tau PET, and temporal-metaROI cortical thickness may be more relevant to AD pathological changes rather than the normal aging process. Additionally, we found females had more astrocyte reactivity but less axonal degeneration than males. Consequently, the age effect and sex difference in plasma GFAP and plasma NfL concentrations should be considered when evaluating AD-related astrocyte reactivity and axonal degeneration. Consistent with previous findings, the *APOE-ε4* carriers showed higher cortical Aβ deposition [61] and less cortical thinning [62] than the *APOE-ε4* non-carriers.

In this study, we defined the community-useful thresholds for plasma Aβ_42_/Aβ_40_ and p-Tau_181_ and evaluated their feasibility in identifying individuals with a high risk of AD based on Aβ PET, tau PET, and MRI image data in a large community-based Chinese aging cohort. Furthermore, this study demonstrated the critical role of astrocyte reactivity in AD progression, particularly in tau aggregation and cortical thinning in AD-signature cortical regions. This supports the usefulness of inflammation (I) biomarkers (such as plasma GFAP) to establish a more accurate diagnosis scheme in the clinic but also provides novel insights into understanding how astrocyte reactivity affects the cortical tau aggregation and cortical thinning in AD. However, all the analyses in this study were conducted based on cross-sectional data, and these findings are correlational in nature. Thus, this study does not establish plasma Aβ_42_/Aβ_40_, plasma p-Tau_181_, or plasma GFAP as causal factors. All we can be sure of is that plasma GFAP seems to have a strong influence on elevated tau aggregation and cortical thinning in AD-signature cortical regions. Additionally, YKL40 may also be an astrocyte-related biomarker, which appears to be related to pathological AD changes in the late stage [44, 63]. Thus, further investigation is critical to confirm the influence of other astrocyte biomarkers in AD progression. Notably, the findings that higher plasma GFAP concentrations may be related to more plasma p-Tau181-related tau tangle aggregation should be validated using an independent cohort with a larger sample size in the future. Finally, the longitudinal data of plasma biomarkers, Aβ PET, tau PET, and MRI images are essential to validate all the cross-sectional findings in the present study.

## Conclusions

In summary, this study established the thresholds of plasma Aβ_42_/Aβ_40_ and plasma p-Tau_181_ measured by the Sioma platform and revealed the biological staging of AD scheme using plasma biomarkers in a Chinese community-based aging cohort. Plasma Aβ_42_/Aβ_40_ positive and plasma p-Tau_181_ positive (A+/T+) individuals showed significant evidence of cortical Aβ deposition, tau aggregation, hippocampal atrophy, and cortical thinning. The first-defined community useful thresholds of plasma Aβ_42_/Aβ_40_ and plasma p-Tau_181_ in the older Chinese Han population offer a significant reference for early AD diagnosis in China, although more validation is still essential. The astrocyte reactivity reflected by plasma GFAP may be a promising “I” biomarker, augmenting the progression of A+/T + individuals defined by plasma Aβ_42_/Aβ_40_ and plasma p-Tau_181_. These findings indicate that the A/T/I profiles may provide more comprehensive information for selecting high AD-risk individuals for clinical trials and highlight the importance of targeting reactive astrogliosis to prevent AD progression.

### Electronic supplementary material

Below is the link to the electronic supplementary material.

Supplementary Material 1

Supplementary Material 2

## Data Availability

The data used in the current study were obtained from the GHABS cohort. Derived data is available from the corresponding author on request by any qualified investigator subject to a data use agreement.

## Abbreviations

Aβ: β-amyloid
A +: Aβ positive
APOE: Apolipoprotein E
β_std_: standardizedβ
CI: Cognitive impaired
ci: confidence interval
CSF: cerebrospinal fluid
CU: cognitively unimpaired
FSP: [18 F]D3FSP
FTP: [18 F]-flortaucipir
FWE: family-wise error
GFAP: Glial fibrillary acidic protein
GHABS: Greater-Bay-Area Healthy Aging Brain Study
IQR: Interquartile range
MCI: Mild cognitive impairment
NfL: Neurofilament light
p-Tau: Phosphorylated tau
rHCV: residual hippocampal volume
ROC: Receiver operating characteristic curve
ROI: Region of interest
SCD: Subjective cognitive decline
SUVR: Standardized uptake value ratio
T +: Tau positive
