# Supplementary material for: Astrocyte reactivity is associated with tau tangle load and cortical thinning in Alzheimer’s disease
Source: Mol Neurodegener. 2024 Jul 30;19:58. doi: 10.1186/s13024-024-00750-8 (PMC11290175; doi:10.1186/s13024-024-00750-8)
Supplement: Supplementary file 1 — Supplementary Material 1 [file 13024_2024_750_MOESM1_ESM.pdf]

Supplementary Material

Supplementary Material...1

Inclusion and exclusion criteria..2

Definition of clinical diagnosis..4

Blood sample collection and processing..4

PET imaging acquisition and preprocessing..5

Cutoffs of Aβ PET FSP SUVR..6

Cutoffs of plasma Aβ42/Aβ40 and plasma p-Tau181...7

Comparisons of plasma biomarkers and neuroimage data between different A/T profiles.11

Inclusion and exclusion criteria

The GHABS cohort recruits men and women between the ages of 55 and 90. The inclusion criteria are as listed:

- Age 55-90 years old (including 55 and 90 years old).
- The score of the Geriatric Depression Scale (GDS) is less than 6 points.
- There is a caregiver who can maintain at least 10 hours of contact per week and can accompany volunteers to the test site for testing.
- Visual and auditory acuity is sufficient for neuropsychological testing. (Including normal corrected vision and hearing)
- Be in good health and are expected to be free of disease interference during the study.
- Volunteers are not pregnant, lactating or have reproductive potential (that is, women must be two years after menopause or undergo sterilization surgery).
- Willingness and ability to participate in longitudinal imaging studies.
- A modified version of the Hachinski Ischemic scores less than or equal to 4.
- Have completed grade 6 education or have good work experience (sufficient to rule out mental retardation).
- Must be able to speak Mandarin fluently.
- Willing to undergo multiple 3T MRI scans and at least two PET scans.
- Agree to collect blood for genomic analysis (including GWAS sequencing and other analyses), AD risk and protective genes such as apolipoprotein E (APOE), klotho, etc., and biological sample storage.
- Agree to collect blood for biomarker detection.
- Agree to share genomic data and biomarker samples.

The exclusion criteria are as follows:

- MRI brain scan screening reveals infection, infarction or other focal lesions or multiple lacunes or lacunes in key memory structures.
- Any volunteers who do not meet the MRI scan requirements, including having a cardiac pacemaker, eyes, skin or metal fragments or foreign bodies in the body.
- Severe depression, bipolar affective disorder described in DSM-IV in the past year.
- Psychotic features, agitation or behavioral problems that may lead to difficulty complying with the protocol content in the past 3 months.
- Currently using medication to treat obsessive-compulsive disorder or attention deficit disorder.
- History of schizophrenia (meeting DSM-IV criteria).
- History of alcohol or drug abuse or dependence within the past 2 years (metting DSM-IV criteria).
- Any major systemic disease or unstable physical condition that may make longitudinal research difficult.
- Clinically significant abnormalities of B12 or TFTs may interfere with the study, low B12 will be excluded.
- Currently using certain psychoactive medications (e.g., certain antidepressants, neuro-depressants, chronic anxiolytics, or sedative-hypnotics). Currently using warfarin or other anticoagulants such as dabigatran, rivaroxaban, and apixaban (except lumbar puncture).
- Use of prohibited drugs.
- Simultaneously participating in other clinical studies involving neuropsychiatry.

Definition of clinical diagnosis

Several cognitive assessments were used for clinical diagnosis. The normal performance of these assessments was defined based on education or age. The Mini-Mental State Examination (MMSE) used cutoff scores as follows: >17 for participants without education, >20 for 1-6 years of education, and <24 for more than 6 years of education. The delayed recall of logical Memory test used cutoff scores as follows: ≥3 for 0-7 years of education, ≥5 for 8-15 years of education, and ≥9 for 16 years of education. As for the activities of daily living (ADL), <23 is normal for participants under 75 years old, while <25 for 75 years and older.

Normal control (NC) participants were normal in MMSE, logical Memory recall, and ADL, and their CDR score was 0. Participants with MCI were normal performance in MMSE while they showed impairment in logical Memory test. Their CDR score was 0.5 with a mandatory requirement of the memory box score being 0.5 or greater, but ADL was normal. Participants with AD dementia were abnormal in MMSE, logical memory and ADL. The CDR score was 0.5 or greater.

Blood sample collection and processing

Volunteers fasted for one night the day before (not less than 6 hours), and blood was drawn in the morning of the next day. The venous blood of the volunteers was drawn into two 10 ml EDTA blood collection tubes and gently inverted and mixed 10-12 times to ensure that the blood and anticoagulant were thoroughly mixed. The mixed blood was placed in an incubator at 4°C and shipped back to the laboratory within 4 hours for subsequent analysis. The blood was centrifuged at 1600g for 10 minutes in a refrigerated centrifuge at 4°C. The upper plasma layer was transferred to several 2 ml centrifuge tubes using a sterile RNase-free pipette tip. To obtain more pure plasma, the separated plasma was centrifuged again at 16000

g for 10 minutes at 4°C, and then the supernatant was aliquoted into several 0.5 ml centrifuge tubes with labels, each with either 100 or 200 μl blood plasma. The aliquots were stored in a -80°C refrigerator for subsequent analysis. After the whole blood was centrifuged in the first step, the buffy coat in the middle layer was gently transferred to the 2640 medium. Then, after density gradient centrifugation, erythrocyte lysis, and centrifugation steps, the isolated peripheral blood mononuclear cell sample was transferred to a 2ml RNase-free centrifuge tubes and stored in a gradient-cooled freezer box at -80°C for subsequent analysis.

PET imaging acquisition and preprocessing

A dedicated head scanning procedure covering the whole brain from vertex to cerebellum was used for imaging. A diagnostic dose CT scan of the brain was acquired beforehand for attenuation correction and fusion localization of PET images. The PET scans were acquired using 3D Listmode on the GE Discovery MI. The field of view (FOV) was 256 mm×256 mm×220 mm, the scanning matrix was 336×336×109, and the voxel size was 1.02 mm×1.02 mm×2.03 mm. All the correction options were selected, and no filter or smooth was used during the reconstruction. A reconstruction offset was applied to ensure that the head was completely in the field of view within the plane. Finally, 4 frames of dynamic images were generated according to 5 min/frame segmentation, with each PET scan corresponding to a 20-minute PET image. The PET images were then preprocessed with the following steps before further analysis: 1) co-registering the 2nd, 3rd, and 4th frames to the 1st frame, respectively; 2) averaging the four frames into one; 3) the averaged frame resliced into a standard AC-PC space (anterior commissure-posterior commissure) with image size = 160×160×96, voxel dimension = 1.5mm×1.5 mm×1.5 mm; 4) smoothing with a uniform Gaussian kernel function with a full-width at half maximum (FWHM) of 6 mm.

Cutoffs of Aβ PET FSP SUVR

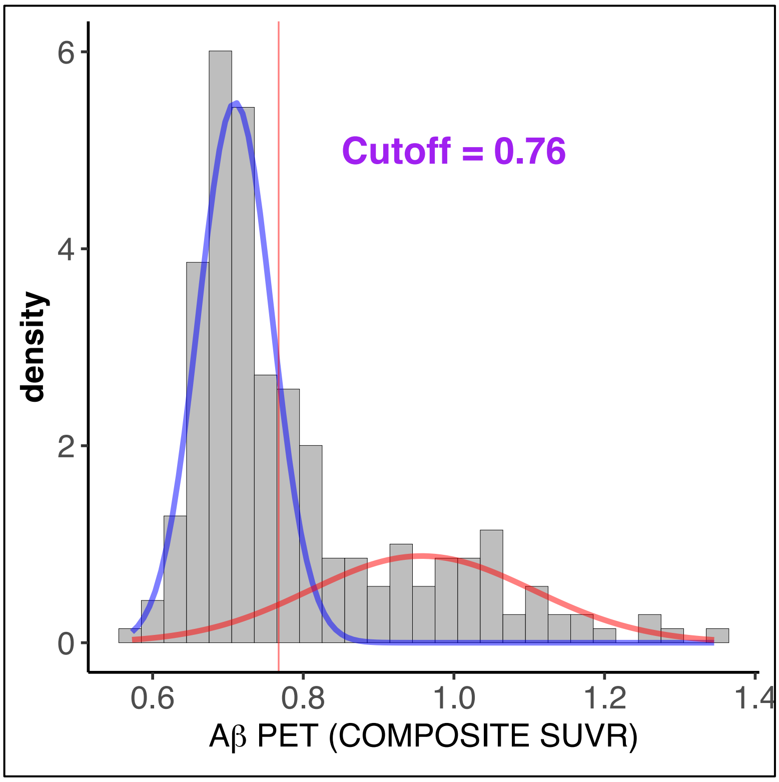

Supplemental Figure 1. *Estimates of two Gaussian distributions of low Aβ (blue curve) and high Aβ (red curve) for COMPOSITE FSP SUVRs of 233 individuals. The red dash vertical line reflects the Aβ+ threshold of COMPOSITE FSP SUVR 0.76, which corresponds to a 90% probability of belonging to the high Aβ distribution.*

Cutoffs of plasma Aβ42/Aβ40 and plasma p-Tau181

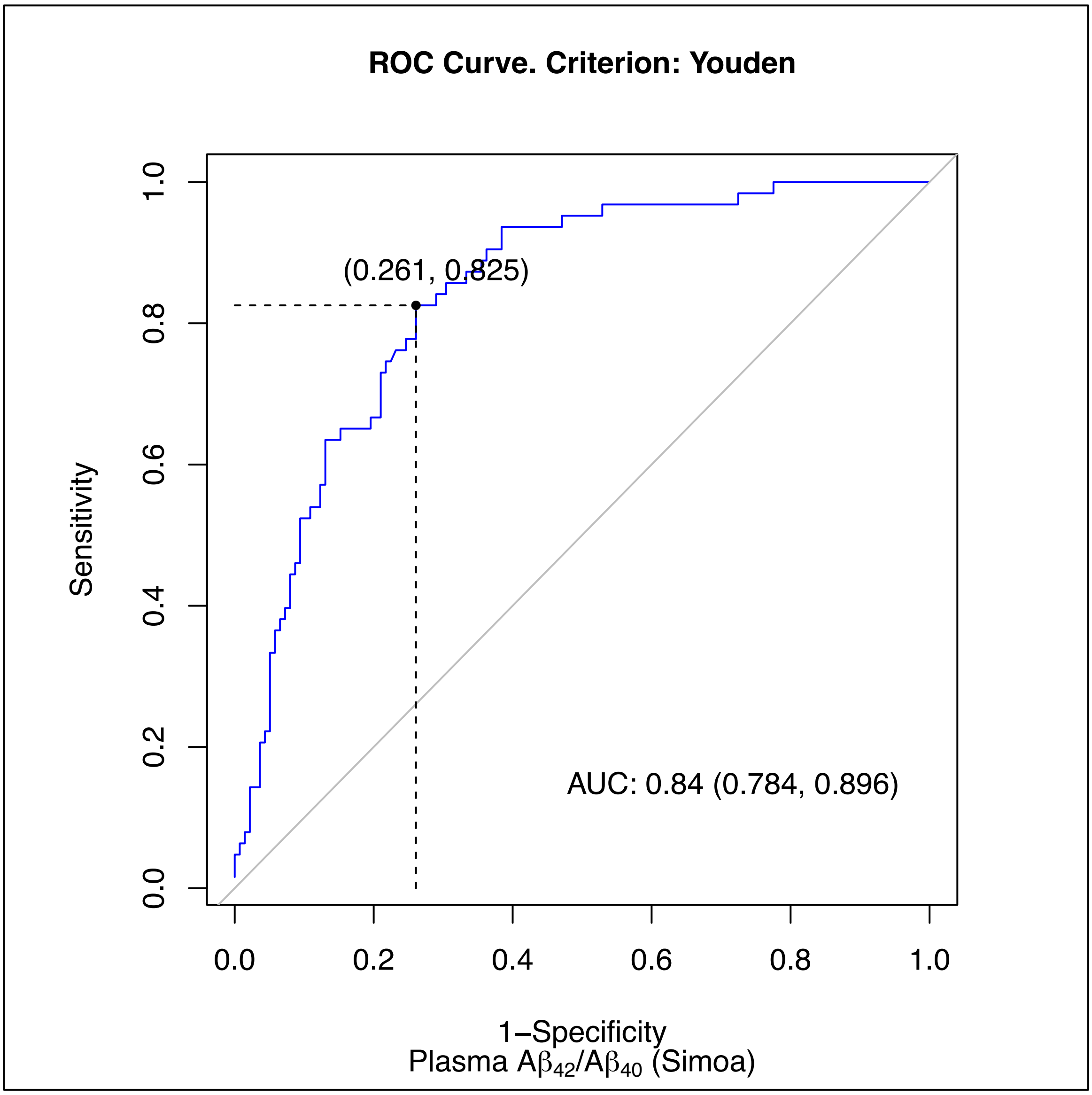

Supplemental figure 2. *The ROC analysis using the Youden index classifying 138 Aβ- cognitively unimpaired (CU) participants and 63 Aβ+ mild cognitive impairment (MCI) and dementia patients as the endpoint to define the cutoff ≤ 0.0609 for plasma $A\beta_{42}/A\beta_{40}$ ratio. AUC: 0.84 (95% CI, 0.83, 0.74).*

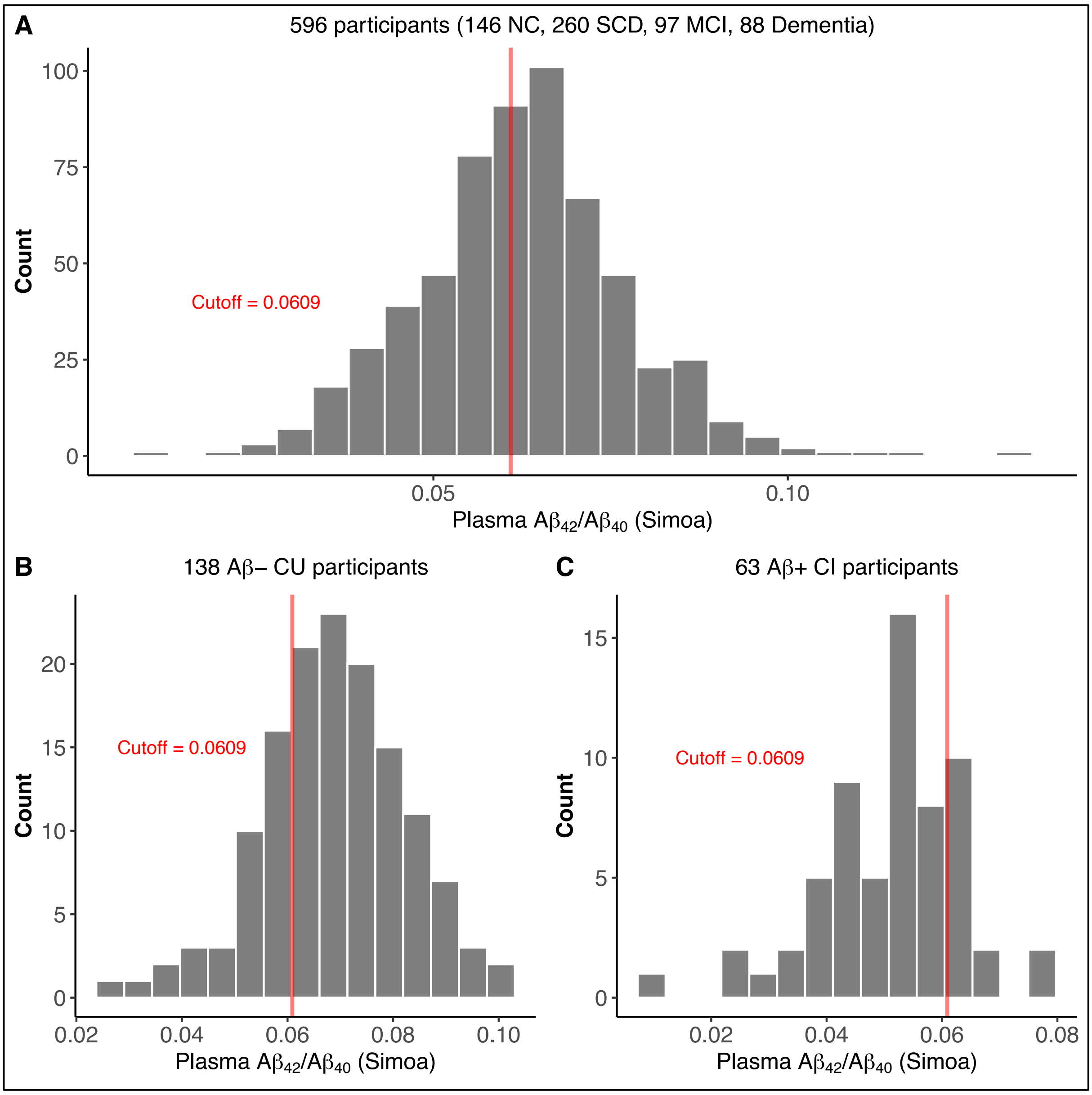

Supplemental figure 3. *Histograms of plasma Aβ$_{42}$/Aβ$_{40}$ for (A) all 596 GHABS participants, (B) 138 Aβ- GHABS CU participants and (C) 63 Aβ+ GHABS MCI and dementia patients. The red dotted line is the cutoff ≤0.0609 for the plasma Aβ$_{42}$/Aβ$_{40}$ ratio.*

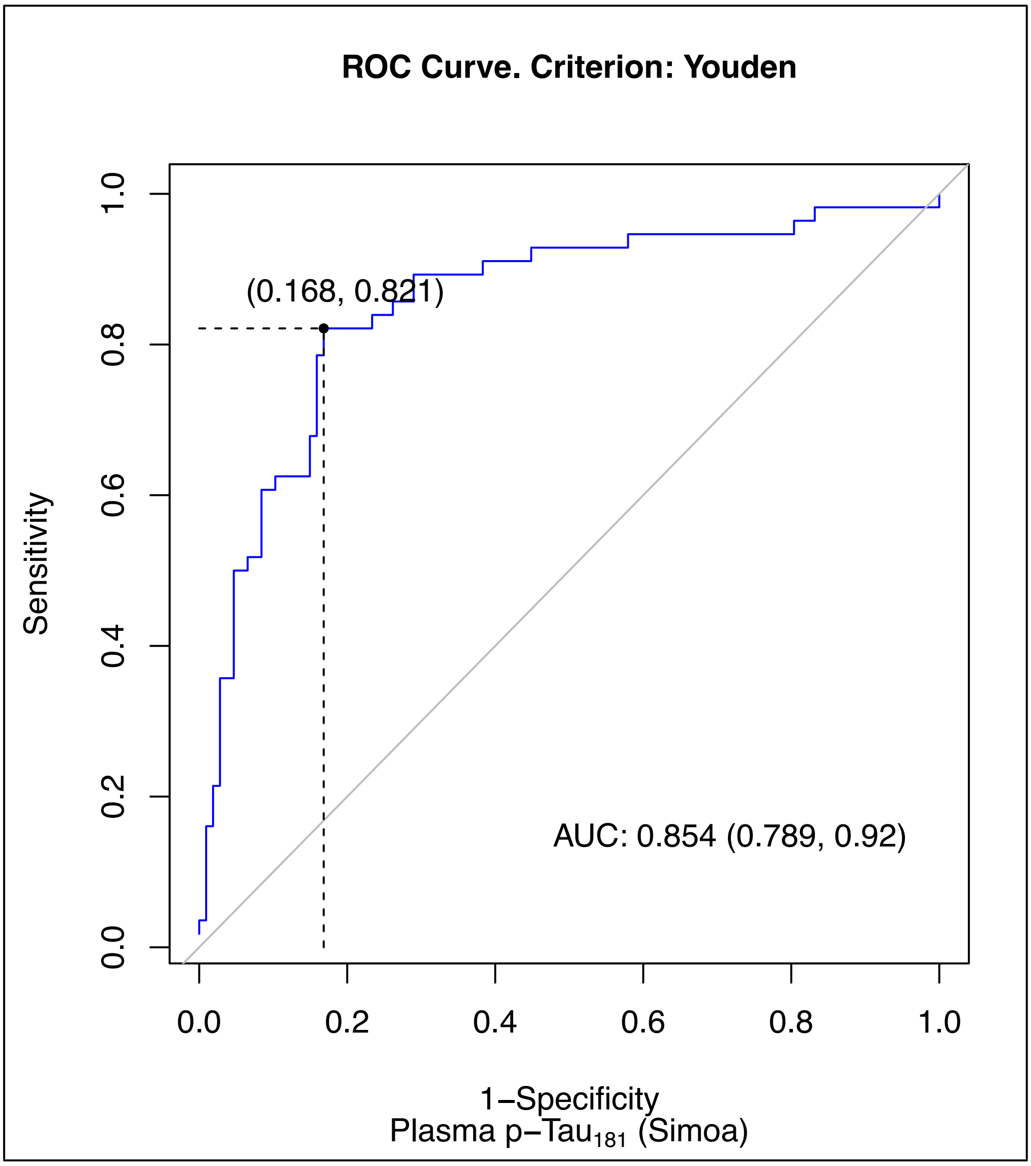

Supplemental figure 4. *The ROC analysis using the Youden index classifying 131 Aβ- GHABS CU participants and 62 Aβ+ GHABS MCI and dementia patients as the endpoint to define the cutoff ≥ 2.418 for plasma p-Tau*$_{181}$*. AUC: 0.85 (95% CI, 0.82, 0.83).*

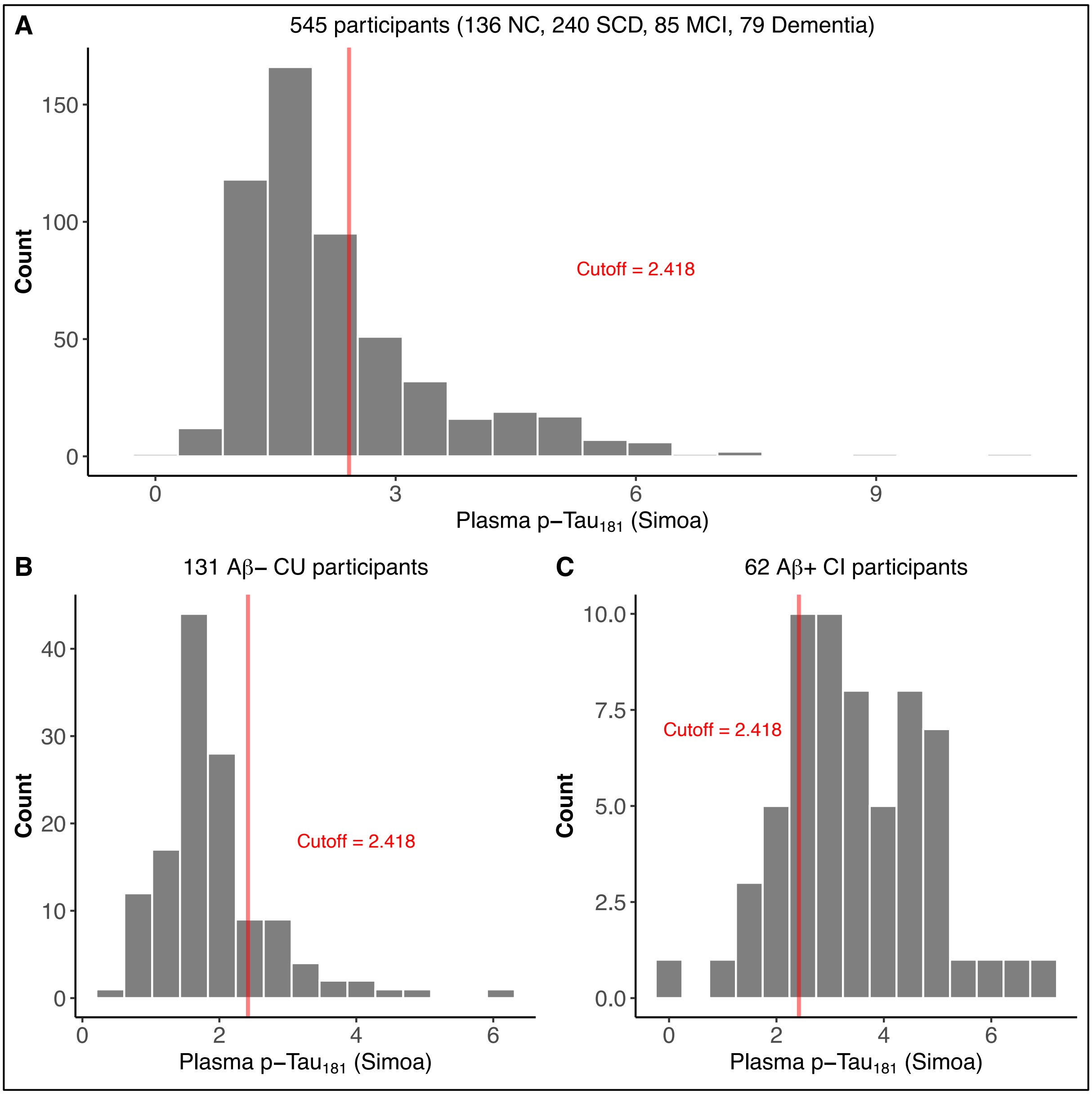

Supplemental figure 5. *Histograms of plasma p-Tau$_{181}$ for (A) all 545 GAHBS participants, (B) 131 Aβ- GHABS CU participants and (C) 62 Aβ+ GHABS MCI and dementia patients. Red dotted line is the 2.418 cutoff for the plasma p-Tau$_{181}$.*

Comparisons of plasma biomarkers and neuroimage data between different A/T profiles

A+T+ individuals showed higher plasma GFAP (Vs. A-/T-: β_{std} = 0.749[95% ci, 0.493, 1.006], $p < 0.001$; Vs. A-/T+: β_{std} = 0.799[95% ci, 0.509, 1.089], $p < 0.001$; Vs. A+/T-: β_{std} = 0.496[95% ci, 0.234, 0.759], $p < 0.001$), plasma NfL (Vs. A-/T-: β_{std} = 0.247[95% ci, 0.005, 0.489], $p = 0.046$; Vs. A-/T+: β_{std} = 0.056[95% ci, -0.218, 0.330], $p = 0.689$; Vs. A+/T-: β_{std} = 0.372[95% ci, 0.124, 0.620], $p = 0.003$), COMPOSITE Aβ PET SUVR (Vs. A-/T-: β_{std} = 0.973[95% ci, 0.666, 1.280], $p < 0.001$; Vs. A-/T+: β_{std} = 0.512[95% ci, 0.167, 0.857], $p = 0.004$; Vs. A+/T-: β_{std} = 0.779[95% ci, 0.444, 1.114], $p < 0.001$), Temporal-metaROI FTP SUVR (Vs. A-/T-: β_{std} = 0.733[95% ci, 0.289, 1.178], $p = 0.001$; Vs. A-/T+: β_{std} = 0.497[95% ci, 0.020, 0.975], $p = 0.041$; Vs. A+/T-: β_{std} = 0.865[95% ci, 0.443, 1.287], $p < 0.001$), and lower rHCV (Vs. A-/T-: β_{std} = -0.577[95% ci, -0.855, -0.299], $p < 0.001$; Vs. A-/T+: β_{std} = -0.358[95% ci, -0.672, -0.044], $p = 0.025$; Vs. A+/T-: β_{std} = -0.474[95% ci, -0.767, -0.181], $p = 0.002$), and Temporal-metaROI cortical thickness (Vs. A-/T-: β_{std} = -0.440[95% ci, -0.760, -0.120], $p = 0.007$; Vs. A-/T+: β_{std} = -0.413[95% ci, -0.774, -0.051], $p = 0.025$; Vs. A+/T-: β_{std} = -0.358[95% ci, -0.695, -0.021], $p = 0.037$) than the A-/T-, A-/T+, and A+/T- groups.

Association of plasma Aβ42/Aβ40, p-Tau181, and GFAP with plasma NfL

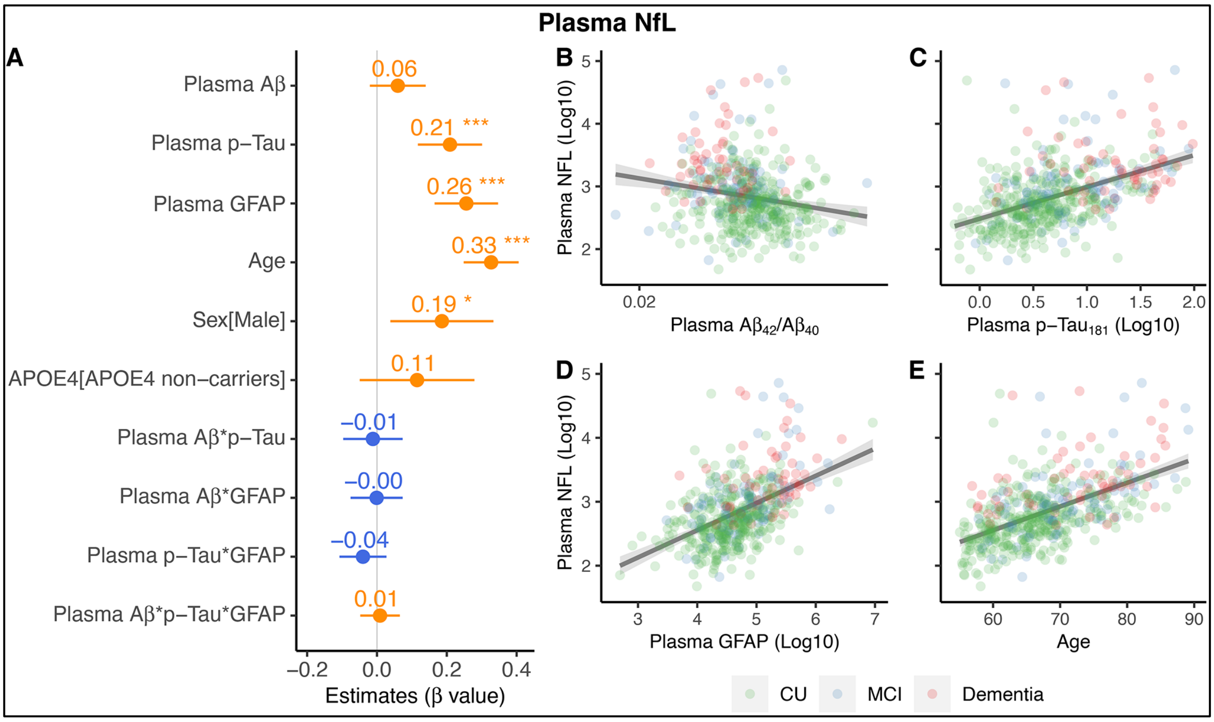

Supplemental figure 6. Association of plasma $A\beta_{42}/A\beta_{40}$ and plasma p-Tau_{181} with plasma NfL.

(A) Independent and interactive association of plasma $A\beta_{42}/A\beta_{40}$, plasma p-Tau_{181}, and plasma GFAP with plasma NfL. Relation of plasma NfL with (B) plasma $A\beta_{42}/A\beta_{40}$, (C) plasma p-Tau_{181}, (D) plasma GFAP, and (E) age. Plasma p-Tau_{181}, plasma GFAP, and plasma NfL were $\log_{10}$ transferred before they were used in the general linear models.

Higher plasma p-Tau_{181} (β_{std} = 0.209[95% ci, 0.117, 0.301], $p < 0.001$) and plasma GFAP concentrations (β_{std} = 0.256[95% ci, 0.165, 0.347], $p < 0.001$), older ages (β_{std} = 0.327[95% ci, 0.248, 0.406], $p < 0.001$), and males (β_{std} = 0.186[95% ci, 0.038, 0.334], p = 0.014) were associated with higher plasma NfL levels (Supplemental figure 6).

Association of plasma $A\beta_{42}/A\beta_{40}$, p-Tau_{181}, and GFAP with Aβ plaques and tau tangles

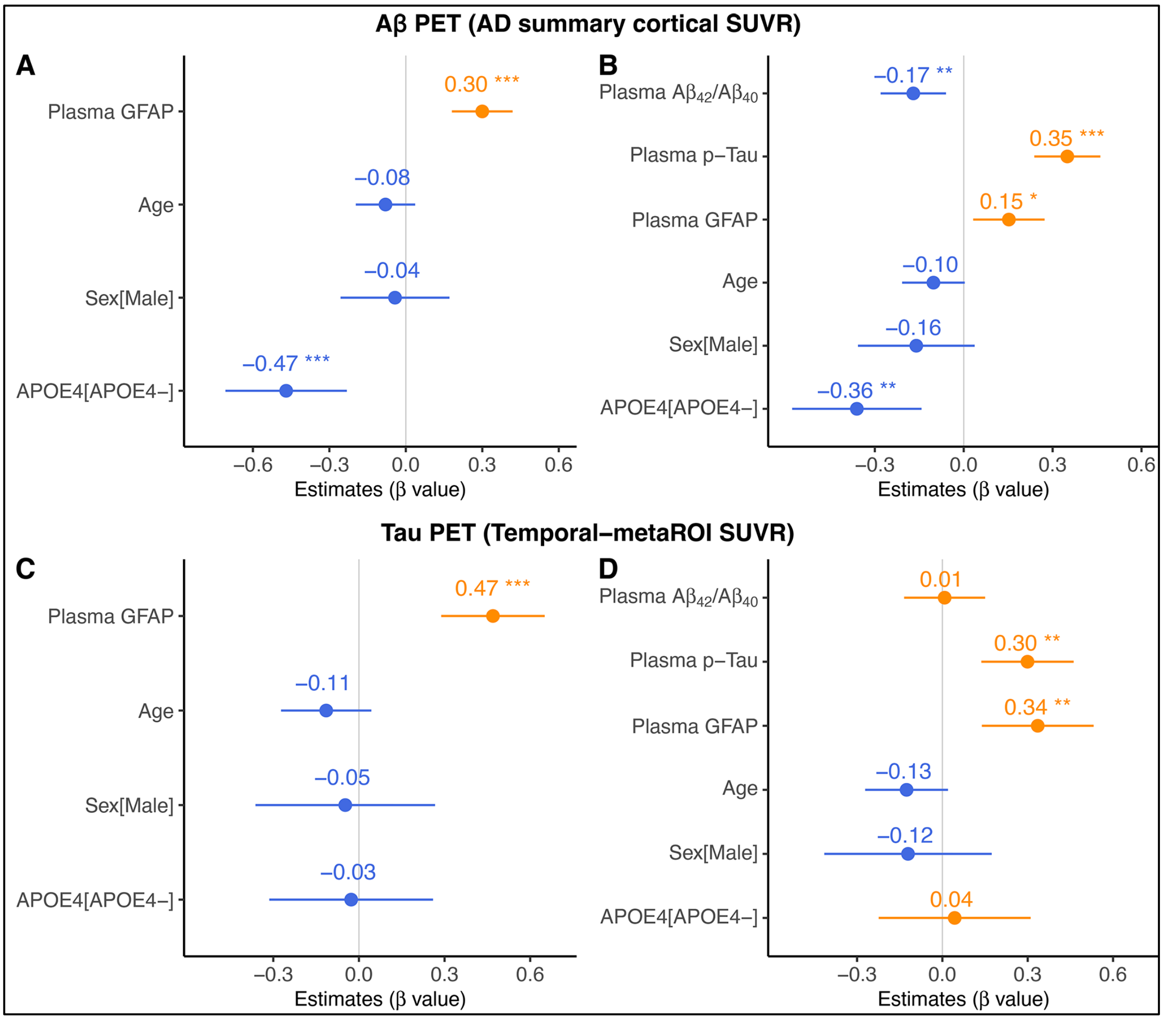

Supplemental figure 7. *Association of plasma GFAP with Aβ PET and tau PET without (A, C) and with (B, D) controlling for plasma Aβ42/Aβ40 and plasma p-Tau181 in the models.*

Association of plasma $A\beta_{42}/A\beta_{40}$, p-Tau_{181}, and GFAP with hippocampal atrophy and cortical thinning

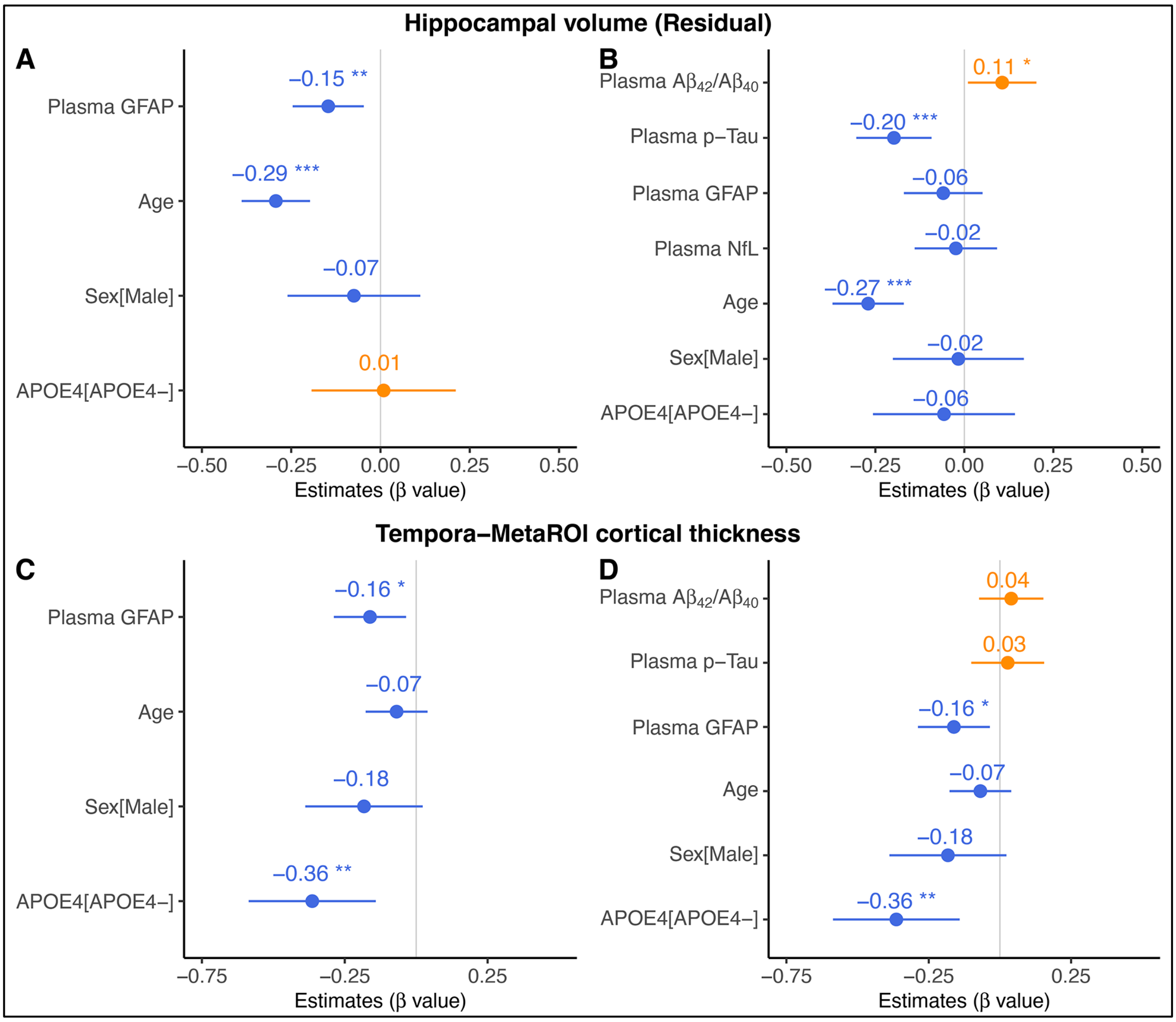

Supplemental figure 8. Association of plasma GFAP with hippocampal atrophy and cortical thinning. *Association of plasma GFAP with residual hippocampal volume (rHCV) and (C-D) temporal-metaROI cortical thickness (A, C) without and (B, D) with controlling for plasma* $A\beta_{42}/A\beta_{40}$*, plasma p-*Tau_{181}*, and. Notably, Plasma p-*Tau_{181} *and plasma GFAP were* log_{10} *transferred before they were used in the general linear models.*

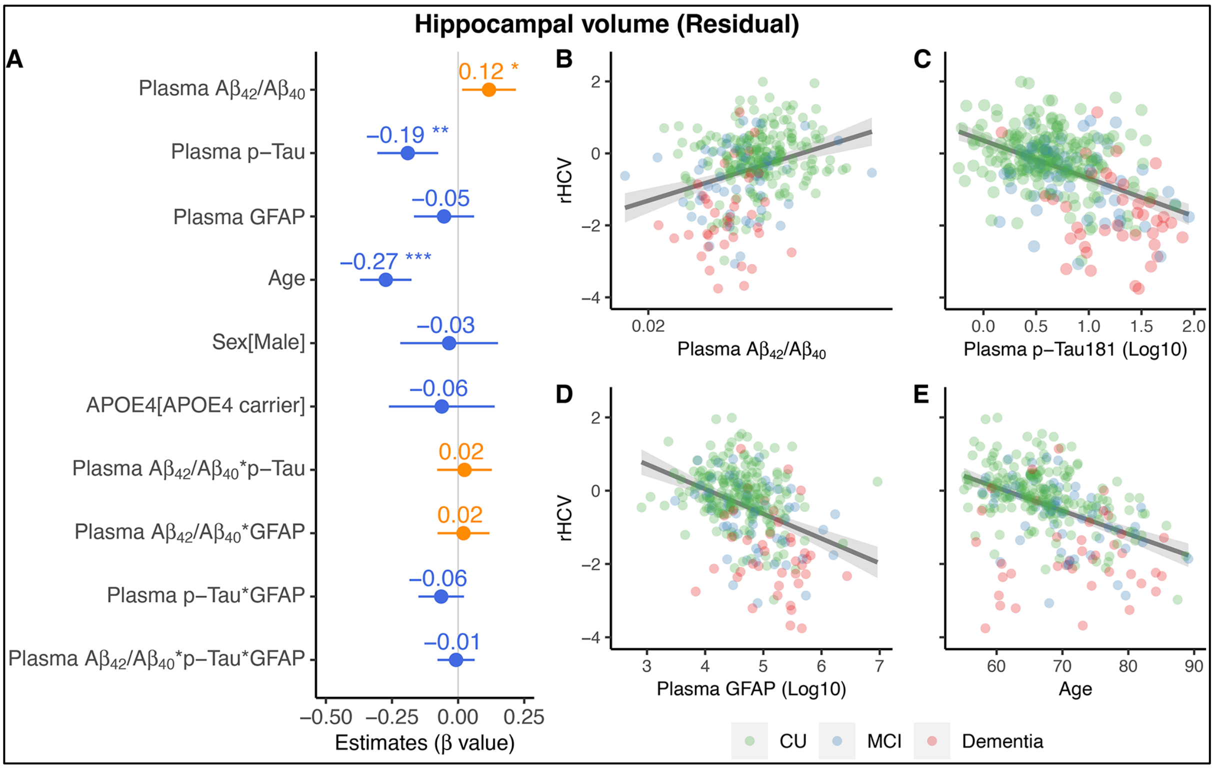

Supplemental figure 9. Association of plasma $A\beta_{42}/A\beta_{40}$, plasma p-Tau_{181}, and plasma GFAP with hippocampal atrophy. *Association of plasma $A\beta_{42}/A\beta_{40}$, plasma p-Tau_{181}, and plasma GFAP with (A-E) residual hippocampal volume (rHCV). Notably, Plasma p-Tau_{181} and plasma GFAP were log_{10} transferred before they were used in the general linear models.*
