# Supplementary material for: Astrocyte reactivity is associated with tau tangle load and cortical thinning in Alzheimer’s disease
Source: Mol Neurodegener. 2024 Jul 30;19:58. doi: 10.1186/s13024-024-00750-8 (PMC11290175; doi:10.1186/s13024-024-00750-8)
Supplement: Supplementary file 2 — Supplementary Material 2 [file 13024_2024_750_MOESM2_ESM.pdf]

Tengfei Guo, PhD
Assistant Professor
Institute of Biomedical Engineering, Shenzhen Bay Laboratory, Peking University Shenzhen Graduate School, Shenzhen, China
Email: tengfei.guo@pku.edu.cn, phone: +86-0755-2684-9264

July 22nd, 2024

Dear Prof. Bu,

We thank you for providing reviews of our manuscript "*Astrocyte reactivity is associated with tau tangle load and cortical thinning in Alzheimer's disease*" (MS ID#: MOND-D-24-00031) again. We have thoroughly addressed the concerns raised by the Editor and replied to him/her in our response letter. Our responses are *italicized* below. The details of our response can be found in the response letter. We hope you can like this version and consider it for publication as a "*Research Article*" in the journal "*Molecular Neurodegeneration*".

Kind regards,
Tengfei Guo and co-authors

Ph.D., Assistant Professor, Institute of Biomedical Engineering

Shenzhen Bay Laboratory, Peking University Shenzhen Graduate School

Address: No.5 Kelian Road; Tower A, GAOKE INTERNATIONAL INNOVATION CENTER, Shenzhen, Guangdong, China 518000

Email: tengfei.guo@pku.edu.cn
